# The hubs of the human connectome are generally implicated in the anatomy of brain disorders

**DOI:** 10.1093/brain/awu132

**Published:** 2014-06-19

**Authors:** Nicolas A. Crossley, Andrea Mechelli, Jessica Scott, Francesco Carletti, Peter T. Fox, Philip McGuire, Edward T. Bullmore

**Affiliations:** 1 Department of Psychosis Studies, Institute of Psychiatry, King’s College London, London SE5 8AF, UK; 2 Research Imaging Institute and Department of Radiology, The University of Texas Health Science Centre at San Antonio, San Antonio, TX 78229, USA; 3 University of Cambridge, Behavioural & Clinical Neuroscience Institute, Department of Psychiatry, Cambridge CB2 0SZ, UK; 4 Cambridgeshire and Peterborough NHS Foundation Trust, Cambridge CB21 5EF, UK; 5 GlaxoSmithKline, ImmunoPsychiatry, Alternative Discovery and Development, Stevenage SG1 2NY, UK

**Keywords:** topology, VBM, graph analysis, rich club, tractography

## Abstract

Brain networks or ‘connectomes’ include a minority of highly connected hub nodes that are functionally valuable, because their topological centrality supports integrative processing and adaptive behaviours. Recent studies also suggest that hubs have higher metabolic demands and longer-distance connections than other brain regions, and therefore could be considered biologically costly. Assuming that hubs thus normally combine both high topological value and high biological cost, we predicted that pathological brain lesions would be concentrated in hub regions. To test this general hypothesis, we first identified the hubs of brain anatomical networks estimated from diffusion tensor imaging data on healthy volunteers (*n* = 56), and showed that computational attacks targeted on hubs disproportionally degraded the efficiency of brain networks compared to random attacks. We then prepared grey matter lesion maps, based on meta-analyses of published magnetic resonance imaging data on more than 20 000 subjects and 26 different brain disorders. Magnetic resonance imaging lesions that were common across all brain disorders were more likely to be located in hubs of the normal brain connectome (*P < *10^−4^, permutation test). Specifically, nine brain disorders had lesions that were significantly more likely to be located in hubs (*P < *0.05, permutation test), including schizophrenia and Alzheimer’s disease. Both these disorders had significantly hub-concentrated lesion distributions, although (almost completely) distinct subsets of cortical hubs were lesioned in each disorder: temporal lobe hubs specifically were associated with higher lesion probability in Alzheimer’s disease, whereas in schizophrenia lesions were concentrated in both frontal and temporal cortical hubs. These results linking pathological lesions to the topological centrality of nodes in the normal diffusion tensor imaging connectome were generally replicated when hubs were defined instead by the meta-analysis of more than 1500 task-related functional neuroimaging studies of healthy volunteers to create a normative functional co-activation network. We conclude that the high cost/high value hubs of human brain networks are more likely to be anatomically abnormal than non-hubs in many (if not all) brain disorders.

## Abstract (precis)

See Sporns (doi:10.1093/brain/awu148) for a scientific commentary on this article.

Brain networks contain a minority of highly connected hub nodes with high topological value and biological cost. Using network analysis of DTI data from healthy volunteers, and meta-analyses of published MRI studies in 26 brain disorders, Crossley *et al*. show that lesions across disorders tend to be concentrated at hubs.

## Introduction

Connectomes or brain networks derived from neuroimaging data have several non-random or complex topological properties (Bullmore and Sporns, 2009), including fat-tailed degree distributions that reflect the existence of a minority of high degree (highly connected) hub nodes (Eguiluz *et al.*, 2005; Achard *et al.*, 2006; Sporns *et al.*, 2007; van den Heuvel and Sporns, 2013). Hubs mediate many of the long-distance connections between brain modules (Zamora-Lopez *et al.*, 2010), and are efficiently interconnected to form a rich club (van den Heuvel and Sporns, 2011). It has been suggested that these topological features are functionally valuable for integrative information processing and adaptive behaviour (van den Heuvel *et al.*, 2012; Crossley *et al.*, 2013). However, the spatial distance of edges connecting hubs to the rest of the network (a proxy measure of wiring cost) is greater than the distance of edges connecting more topologically peripheral nodes (van den Heuvel *et al.*, 2012; Alexander-Bloch *et al.*, 2013). As such, hubs appear to transgress the parsimonious drive to minimize wiring cost (Kaiser and Hilgetag, 2006), which explains several other organizational features of brain networks (Chen *et al.*, 2006). Recent studies have also shown that hubs, especially in the cortex, have higher rates of cerebral blood flow, aerobic glycolysis and oxidative glucose metabolism (Vaishnavi *et al.*, 2010; Liang *et al.*, 2013; Tomasi *et al.*, 2013). Arguably, this combination of higher metabolic rate and longer connection distance could be summarized by saying hubs are ‘biologically costly’.

Comparable high cost / high value properties of network hubs have been described in brains of other species (Iturria-Medina *et al.*, 2010; Harriger *et al.*, 2012; Towlson *et al.*, 2013), suggesting that an economical trade-off between topological value and biological cost may be a general and scale-invariant selection pressure on the formation of nervous systems (Bullmore and Sporns, 2012; Vertes *et al.*, 2012).

Conceptualizing the brain as a network (the ‘connectome’) (Sporns *et al.*, 2005) has potentially important implications for understanding clinical brain disorders (Bullmore and Sporns, 2012; Fornito *et al.*, 2012; Menon, 2013; Rubinov and Bullmore, 2013; van den Heuvel and Sporns, 2013). For example, cortical hubs have been suggested to be critical regions in Alzheimer’s disease, concentrating most of the amyloid-β deposition (Buckner *et al.*, 2009). Other studies have highlighted the role of topological modularity in the anatomy of brain disorders, showing that focal grey matter abnormalities in neurodegenerative disorders are not randomly located with respect to the modular organization of the normal brain network (Seeley *et al.*, 2009). Furthermore, focal brain lesions affecting nodes involved in intermodular connections cause important reconfiguration of the whole network (Gratton *et al.*, 2012). As most studies have focused on neurodegenerative disorders, a number of disease-specific mechanisms have been proposed to account for the observed relationships between neuropathology and brain network organization (Zhou *et al.*, 2012). For example, trans-synaptic transmission of a pathogenic agent has been suggested to explain the modular distribution of cortical abnormalities (Prusiner, 1984; Pearson *et al.*, 1985; Raj *et al.*, 2012); and this mechanism could also explain higher lesion concentration in hubs (Zhou *et al.*, 2012). It has also been proposed that hubs may have greater susceptibility to oxidative stress, due to their higher rates of metabolic activity (Buckner *et al.*, 2009; Saxena and Caroni, 2011); and a computational model of this pathological mechanism mirrored several neurophysiological aspects of Alzheimer’s disease (de Haan *et al.*, 2012).

Here we investigate a more general theory of the relationship between the topology of the connectome and the anatomy of brain disorders, particularly focusing on the role of hubs. We reasoned that the high cost/high value hubs of the connectome will be particularly crucial for brain disorders for two main reasons. First, their topologically central role could mean that pathological attack on a hub will have a disproportionate impact on the network’s global efficiency of information processing (Albert *et al.*, 2000); and thus be more likely to lead to clinical symptoms such as impairment of cognitive functions, that normally depend on integrative network processes. Second, their topological centrality and high biological cost could make hubs particularly vulnerable to a wide range of pathogenic factors. The main distinction of this theory is its generality: we predicted that any brain disorder, irrespective of its particular aetiology, was likely to impact preferentially on normal brain network hubs.

## Materials and methods

### Diffusion tensor imaging and anatomical network construction

Diffusion tensor imaging (DTI) data from 56 healthy subjects (mean age 32 years, 18 female) were acquired on a 1.5 T GE SIGNA NVi scanner (General Electric) using a cardiac-gated single-shot echo-planar sequence with the following parameters: echo time = 107 ms, repetition time = 15 R-R intervals, 60 contiguous 2.5 mm slices, matrix size 96 × 96 over a 24 cm field of view. Data were zero-filled and reconstructed to a 128 × 128 matrix with a final voxel size of 1.875 × 1.875 × 2.5 mm^3^. Sixty-four diffusion-weighted images (*b* =1300 s mm^−2^) with distributed diffusion sensitization directions and seven images with no diffusion gradients were acquired. Image preprocessing included correction for eddy currents and subject motion, normalization into standardized space using an appropriate B-matrix rotation on the diffusion gradients (Leemans and Jones, 2009), before robust estimation of the tensor model (RESTORE) (Chang *et al.*, 2005). Whole brain deterministic tractography was performed (Basser *et al.*, 2000), with streamlines terminating if encountering a fractional anisotropy threshold <0.2 or a maximum deflection angle of 30°. The whole brain (excluding the cerebellum) was parcellated into 401 similarly-sized regions, respecting anatomical landmarks (Tzourio-Mazoyer *et al.*, 2002; Zalesky *et al.*, 2010). We then calculated the number of streamlines connecting all pairs of regions for every subject, and built a group binary network by drawing an edge when the number of streamlines connecting two regions across subjects was significantly different from zero at *P < *0.01 [rank sum test, false discovery rate (FDR) corrected]. By using a template with similarly-sized regions, we avoided any bias introduced by larger regions having more connections or greater probability of grey matter lesions (Supplementary Fig. 1).

### Topological and spatial measures of the diffusion tensor imaging network

Several graph analytic measures were estimated on the binary network built from the DTI data, including degree (number of connections) and betweenness centrality of each nodal region; global efficiency, rich club and modularity of the network, and participation coefficient of each node in the context of this modular structure (Rubinov and Sporns, 2010). See the online Supplementary material for more detail on graph theoretical metrics. We also estimated the connection distance between each pair of brain regions by measuring the Euclidean distance between their centroids in stereotactic space.

To investigate the resilience of the network, we modelled two modes of attack *in silico*: random and targeted attack. Analysis of resilience to random attack consisted of monitoring the global efficiency of the remaining network after deleting an increasing number of randomly selected nodes (1000 iterations). Targeted attack was performed by deleting nodes in order of decreasing degree, i.e. attacking the highest degree hub first. Change in global efficiency after each node was deleted was expressed as a percentage of the intact network’s global efficiency. We repeated this same procedure but deleting edges rather than nodes: edges were deleted at random or in order of decreasing connection distance, i.e. attacking the longest edge first.

### Meta-analysis of anatomical (voxel-based morphometry) studies of clinical disorders

We included structural MRI studies analysed using voxel-based morphometry (VBM) that reported significant grey matter volume or density reductions in patients with any neurological or psychiatric disorder compared to healthy volunteers. Eligible studies had been published in English language journals, before the date of the search (May 2012), and reported coordinates of grey matter changes in standard stereotactic space. We tried to be as inclusive as possible, and performed 93 different searches in PubMed using terms related to disorders as described in Chapters V and VI of the 10th Edition of the International Classification of Disorders (ICD-10, 2010). We also searched the BrainMap database (Fox and Lancaster, 2002; Fox *et al.*, 2005; Laird *et al.*, 2005) and updated this open access database with the results of any additional primary studies identified by the PubMed searches, so that the complete set of primary coordinates used for the meta-analysis is accessible at http://www.brainmap.org (see Supplementary material and Supplementary Fig. 2 for details).

For each primary study, we identified coordinates of grey matter volume (or density) reduction in patients that were reported as statistically significant at a threshold of *P < *0.05 whole-brain corrected or *P < *0.001 uncorrected. Coordinates reported in Talairach space were transformed into the stereotactic space of the MNI atlas (Lancaster *et al.*, 2007). We used the anatomical likelihood estimation (ALE) method of meta-analysis (Eickhoff *et al.*, 2009) to identify locations of significant grey matter deficits or lesions ‘on average’ over a given set of primary studies with *P* < 0.05 (FDR corrected) and a cluster size threshold of 200 mm^3^. Briefly, anatomical likelihood estimation models peak lesion coordinates using 3D Gaussian kernels, with their width defined according to the sample size included. The extent of overlap of the modelled lesions across studies is compared to null models based on the same number of lesions but randomly distributed in the brain.

Using these methods we created a ‘disorder specific’ meta-analytic map of grey matter lesions for the set of primary studies reporting case-control differences on each of the 26 disorders. We also created ‘disorder general’ lesion maps by meta-analysis of a sample of primary studies representative of all 26 disorders or a large number of them. To avoid the disorder general maps being biased by the most frequently studied disorders, which also tended to be studied in the largest primary samples, we included the same number of studies (*n* = 7) per disorder, by randomly sampling a subset of seven primary studies of those disorders, which had been the focus of more than seven published VBM studies. Additionally we corrected the precision of each primary study for its sample size, to mitigate the disproportionate influence of a few large-sample primary studies on the meta-analytic results (Supplementary material).

To assess the robustness of our results, we constructed two alternative, disorder-general VBM lesion maps: (i) excluding neurodegenerative disorders (amyotrophic lateral sclerosis, Alzheimer’s disease, frontotemporal dementia, Huntington’s disease, progressive supranuclear palsy, Parkinson’s disease and dementia in Parkinson’s), for which hub vulnerability has been previously noted and disease-specific hypotheses suggested (Buckner *et al.*, 2009; Zhou *et al.*, 2012); and (ii) based on a smaller subset of more frequently studied disorders (16 disorders for each of which at least nine VBM studies had been published).

### Relating topologically central hubs to grey matter lesions

To relate the topological properties of the normative connectome to meta-analytically derived grey matter lesions, we first assumed that the degree of a voxel in the meta-analytic lesion maps was equivalent to the degree of the regional node of the DTI network in which it was located. We then used logistic regression to relate the binary status of each voxel (lesion or non-lesion) to the continuous independent variable of its degree defined by the DTI connectome. We used permutation testing to assign *P*-values to the logistic regression coefficients. To do this we compared the observed logistic regression coefficient for each voxel to a permutation distribution of coefficients estimated by fitting the same logistic regression model after randomly permuting the assignment of degrees to regional nodes 10 000 times. Similarly, we compared the difference between the median degree of the lesion and non-lesion voxels to a null distribution based on permutation of the regional degrees.

For the maps of each individual disorder, we used bootstrapping to construct 95% confidence intervals (CIs) for the estimated difference in degree of lesion and non-lesion voxels. We thus repeated the VBM meta-analysis for each disorder 100 times, including the same number of studies randomly sampled with replacement (bootstrap), and calculated the difference between the median degree of the lesion and non-lesion voxels in each of the bootstrapped maps.

### Functional co-activation network

To explore the relationship between VBM lesions and network hubs in a different (functional) network, we used the network of functionally co-activated brain regions, as previously reported in detail (Crossley *et al.*, 2013). Briefly, this network was based on meta-analysis of 1641 task-related neuroimaging studies on healthy volunteers performing a wide range of experimental tasks. The activation coordinates were mapped to a parcellation template image comprising 638 similarly sized regions. Two regional nodes defined by this template were connected by an edge if they were significantly co-activated by experimental tasks. We used the Jaccard index as a measure of co-activation for each pair of regions, which is the number of studies activating both regions divided by the number of studies activating either one of them. Edges were defined probabilistically (*P < *0.01, FDR corrected) and weighted by the corresponding Jaccard index. This co-activation network has several topological and physical properties in common with resting state functional MRI networks (Crossley *et al.*, 2013). We here used weighted degree as the primary measure of centrality (defined as the sum of the Jaccard indices weighting each of the edges to a node). Results were substantially unchanged if we analysed an unweighted version of the network and used binary degree as the measure of centrality.

## Results

### Characteristics of the normal human brain (diffusion tensor imaging) connectome

**Figure 1:**
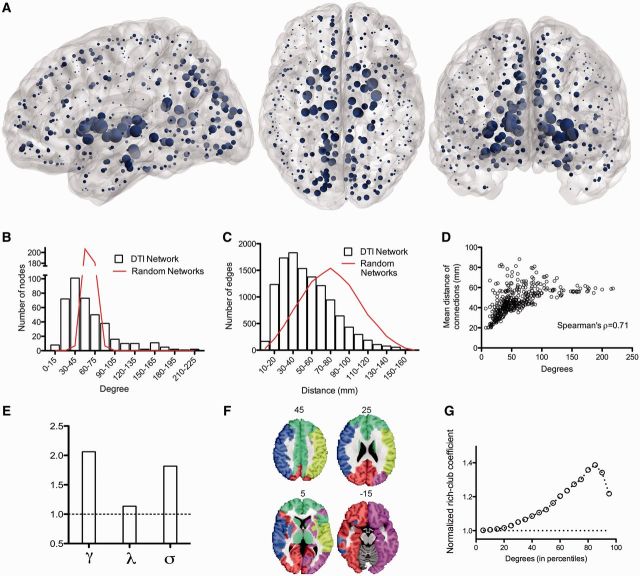
Topological characteristics of the normal brain anatomical network (DTI connectome). (**A**) Nodes of the normal DTI network in anatomical space; the size of each node is proportional to its degree. (**B**) Fat-tailed degree distribution of DTI network (histogram) indicating higher probability of hubs than in a random (Erdös-Rényi) graph (red line). (**C**) Distance distribution of DTI networks (histogram) and of random graphs matched for degree distribution (red line). (**D**) Scatterplot of degree versus mean connection distance in the DTI network. (**E**) Small-world properties of the network (γ = normalized clustering coefficient; λ = normalized path length; σ = ratio of γ to λ; dotted line = 1, the expected value of all these metrics in a random graph). (**F**) Modular decomposition of the DTI network. (**G**) Plot of the normalized rich club coefficient (*y*-axis) as a function of degree threshold (*x*-axis) used to define the rich club; dotted line = 1, the expected value of the normalized rich-club coefficient in a random network with the same degree distribution as the DTI connectome.

The DTI network was a binary graph comprising a single connected cluster of 401 nodes with a connection density of 14.8% (edge-wise probability of false positive error *P < *0.01, FDR corrected). It had a fat-tailed degree distribution, following an exponentially-truncated power law (Clauset *et al.*, 2009), indicating the presence of several high degree hub nodes (Fig. 1B). The top 5% most connected nodes were anatomically located in the posterior cingulate cortex, thalamus, putamen, and hippocampus, as well as parts of the right occipital cortex and left globus pallidus (Fig. 1A).

The network had a small-world organization (Watts and Strogatz, 1998), with a higher clustering coefficient than null models with similar degree distribution (γ = 2.07, 95% CI: 2.05–2.08), whereas its characteristic path length was not much greater than comparable random graphs (λ = 1.135, 95% CI: 1.134–1.136; Fig. 1E). The connectome had a modular community structure [(Newman, 2006), *Q* = 0.38, five modules, Fig. 1F], with hubs mediating many of the intermodular connections (Guimera and Nunes Amaral, 2005) (participation coefficients ranging from 0.63 to 0.75 for the top 5% hubs). The size of the modules explained a low percentage of the variance of nodal degree (7%); whereas degree and participation coefficient were positively correlated and shared ∼34% of their variance (Power *et al.*, 2013). Hubs were also efficiently interconnected with each other to form a rich club (Fig. 1G). All of these results replicate previously published data on independent samples (Hagmann *et al.*, 2008; Gong *et al.*, 2009; van den Heuvel and Sporns, 2011) indicating the replicability of DTI connectomics and establishing the concurrent validity of this connectome as a basis for further analysis.

The wiring cost of the DTI network, as measured by the median Euclidean distance between connected nodes, was reduced compared to that of a random network (*P < *0.001, permutation test); but the distance distribution was fat-tailed indicating some unusually long distance connections (Fig. 1C). In line with previous studies (van den Heuvel and Sporns, 2011; Alexander-Bloch *et al.*, 2013), long-range connections were concentrated on high degree nodes, and there was a significant positive correlation between nodal degree and the mean connection distance of the edges linking each node to the rest of the network (Spearman’s ρ = 0.71, Fig. 1D).

### Resilience of the diffusion tensor imaging connectome to computational attack

**Figure 2:**
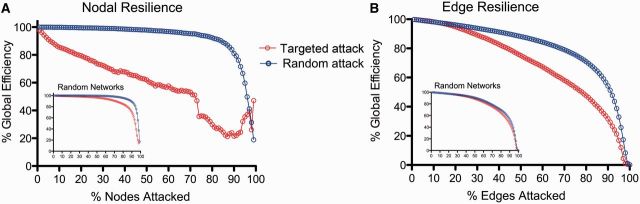
Computational attacks and the resilience of the DTI connectome. (**A**) Plot of global efficiency of the DTI network versus percentage of nodes deleted. When nodes are deleted randomly the efficiency of the network is approximately as resilient as a random (Erdös-Rényi) graph (*inset*); when high degree nodes are targeted (deleted in order of decreasing degree) the efficiency of the network degrades more rapidly than a random graph. (**B**) Plot of global efficiency of the DTI network versus percentage of edges deleted. The efficiency of the DTI network degrades faster than a random graph when the longer distance edges are targeted (deleted in order of decreasing connection distance).

We investigated the resilience of this network to computational attack, by deleting individual nodes or edges and studying the resulting degradation of the network’s global efficiency (Albert *et al.*, 2000). When nodes were attacked randomly, the connectome proved to be almost as resilient as a random graph. However, targeted attack on high degree nodes caused the global efficiency of the brain network to deteriorate more rapidly than the efficiency of a random graph (Fig. 2A). Similarly, the brain network was relatively resilient to attacking edges chosen at random, but targeting long distance edges led to a more rapid deterioration of global efficiency (Fig. 2B). In other words, high degree hub nodes, and the longer distance edges that tended to connect them to the rest of the network, rendered the topological efficiency of the brain network especially vulnerable to targeted attack.

### Relationship of diffusion tensor imaging connectome hubs to anatomical locations of MRI lesions

We then explored the relationship between hubs and lesions using data from MRI studies of 26 neurological and psychiatric disorders. We conducted a systematic, quantitative meta-analysis of the published literature of 392 VBM case-control studies including a total of 9874 patients and 11 502 healthy volunteers (Table 1; citation details of the primary studies are provided in the Supplementary material).

**Table 1:** Disorders included in the meta-analysis of grey matter lesions based on previously published voxel-based morphometry (VBM) studies

Disorder	Number of VBM studies included	Number of patients	Number of healthy controls
Attention deficit hyperactivity disorder	13	363	331
Amyotrophic lateral sclerosis	8	132	146
Anorexia nervosa	10	156	207
Asperger’s syndrome	9	163	209
Autism (pervasive developmental disorder excluding Asperger’s syndrome)	12	330	331
Bipolar affective disorder	18	479	630
Chronic pain	13	305	326
Dementia in Alzheimer’s disease	36	765	1211
Dementia in Parkinson’s disease	10	192	228
Depressive disorder	24	883	1015
Developmental dyslexia	8	121	122
Dystonia	10	219	244
Frontotemporal dementia	37	508	660
Hereditary ataxia	15	202	223
Huntington’s disease	9	227	193
Juvenile myoclonic epilepsy	7	220	218
Multiple sclerosis	11	499	353
Obsessive-compulsive disorder	14	425	431
Obstructive sleep apnoea	7	177	268
Panic disorder	7	142	133
Parkinson’s disease	17	515	411
Progressive supranuclear palsy	7	108	182
Post traumatic stress disorder	14	232	327
Schizophrenia	51	1925	2133
Temporal lobe epilepsy – left	14	339	597
Temporal lobe epilepsy – right	10	247	373
Total	392	9874	11 502

**Figure 3:**
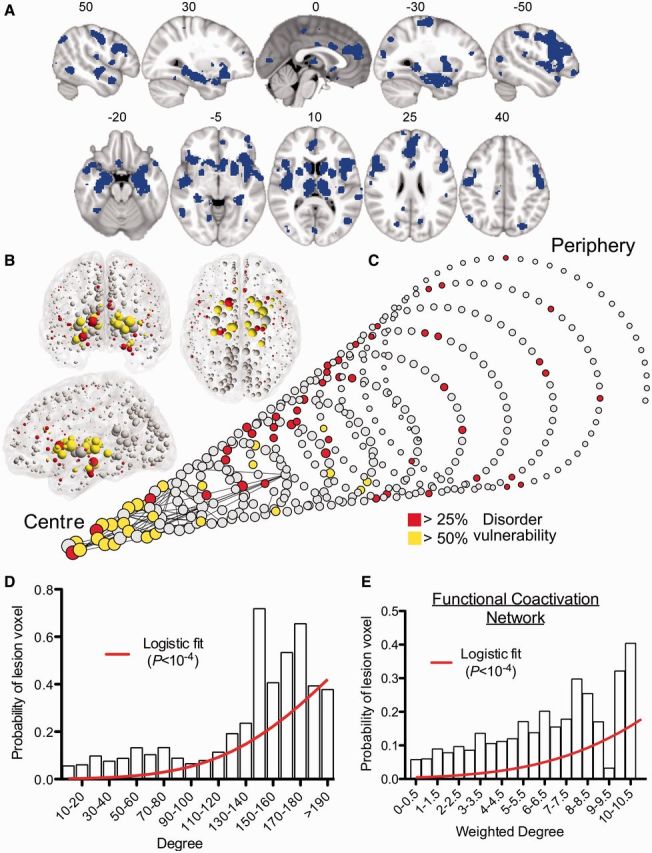
MRI lesions identified meta-analytically from the primary literature on 26 clinical brain disorders impact preferentially on the hubs of the normal connectome. (**A**) A meta-analytic map of multiple cortical and subcortical grey matter MRI lesions that were significantly abnormal ‘on average’ over 26 specific disorders. (**B**) Nodes of the normative DTI connectome, represented in anatomical space, and (**C**) in a spiral, where nodes of similar degree are arranged in the same circle, and the different circumferences arranged so that the tip of the spiral has the highest degree hub nodes, while the base the most peripheral nodes. Nodes are sized in proportion to their degree, and coloured according to the proportion of voxels which are generically lesioned, i.e. the percentage of lesion voxels each node comprises. The strongest 0.1% of edges between nodes, which highlight pairs of nodes with consistently high number of streamlines interconnecting them, are shown for illustrative purposes. (**D**) Plot of the probability of lesion voxels (*y*-axis) versus the degree of DTI connectome nodes (*x*-axis). The red line is a fitted logistic regression model. (**E**) Plot of the probability of lesion voxels (*y*-axis) versus the degree of the functional co-activation network nodes (*x*-axis). The red line is a fitted logistic regression model.

We first combined a balanced subset of these primary studies (including seven studies per each of the 26 disorders; see ‘Materials and methods’ section) into a single map representing brain regions that were, on average, significantly different between patients and healthy volunteers across all disorders. These disorder-general MRI abnormalities of grey matter were located in bilateral thalamus and striatum (putamen and caudate); bilateral hippocampus, insula and superior temporal gyrus; bilateral anterior cingulate cortex, dorsolateral prefrontal cortex and motor cortex; and bilateral superior parietal cortex (Fig. 3A and Supplementary Table 1 for anatomical details). Each voxel in this meta-analytic map could thus be categorized as ‘lesion’ or ‘non-lesion’.

We then aligned the nodes of the normative (DTI) connectome to the coordinates of the meta-analytic lesion map so that each voxel in the lesion map could be assigned the degree of the regional node in which it was anatomically located. Using a simple logistic regression model, we demonstrated that the probability of a voxel being lesioned was significantly related to its nodal degree (*P < *10^−4^, permutation test; Fig. 3B–D). The fitted logistic model predicted a 1.4% increase in the probability that a voxel would be lesioned for every unit increase in degree (each additional connection) of its regional node. Confirming the association between normative network hubs and grey matter lesions, the median degree of lesion voxels (60) was significantly greater than the median degree of non-lesion voxels (47; *P < *10^−4^, permutation test). Another way of looking at these results is to recall that hubs in the DTI connectome form a highly interconnected rich club. We found that 22.5% of the voxels represented by rich-club nodes and 9.8% of voxels represented by peripheral nodes (non rich-club members) were defined as lesion voxels by the VBM meta-analysis, and this difference was statistically significant (*P < *10^−4^, permutation test).

**Figure 4:**
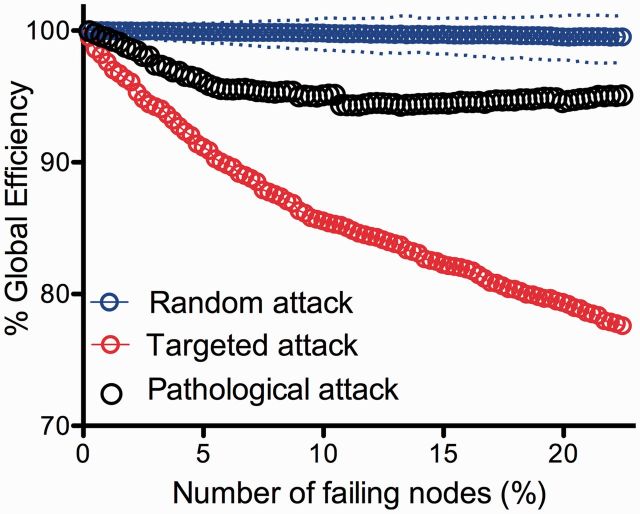
Modelling pathological attack on the connectome. Plot of the global efficiency of the DTI network versus percentage of nodes deleted. Note that the global efficiency deteriorates significantly faster in pathological attacks compared to random attack, but not to the extent of targeted attacks on hubs.

To relate these findings to our computational results on simulated attacks on the network, we modelled pathological attack *in silico*. We first assumed that regions with >20% lesion voxels would not function properly, and therefore we deleted them from the DTI connectome in decreasing order of the proportion of lesion voxels they represented. In other words, the regional node representing the highest proportion of lesion voxels was deleted first and then less-lesioned nodes were incrementally deleted. The global efficiency of the DTI network was degraded by this pathologically targeted attack to a greater extent than by random deletion of nodes; but the connectome was more resilient to pathological attack than it was to attack on hubs (Fig. 4).

**Figure 5:**
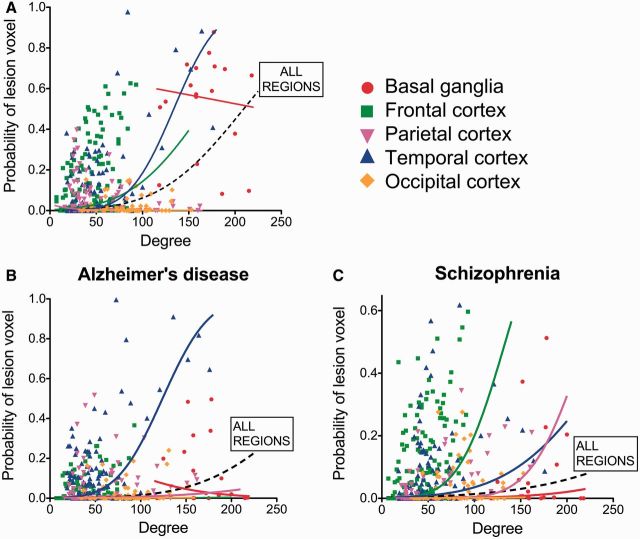
Degree and probability of lesion in anatomical subnetworks. Probability of a ‘voxel lesion’ in each of the 401 regions of the DTI template for (**A**) the disorder-general VBM meta-analysis, as well as for (**B**) Alzheimer’s disease and (**C**) schizophrenia meta-analysis. Nodes have been colour-coded according to their anatomical (lobar) location, and logistic regression lines for each subgroup and for all the regions together are shown.

We also explored the relationship between hubs and lesions in anatomically defined subsets of brain regions: basal ganglia, frontal, parietal, temporal and occipital cortex (Fig. 5A). We found a significant relationship between nodal degree and lesion probability within frontal regions (1.6% increase in lesion probability per unit increase in degree; *P < *0.007, permutation tests) and temporal regions (2.7%, *P < *10^−4^*).* These results in the temporal lobe remained significant when excluding the hippocampus and amygdala from the analysis (1.9%, *P* < 0.005). Parietal regions showed the opposite trend—of lesions being concentrated in low degree nodes (2% decrease in lesion probability per increase in degree, *P < *0.002), although this was not replicated when using the functional co-activation network (see below).

### Relationship of diffusion tensor imaging connectome hubs to the anatomy of specific disorders

**Figure 6:**
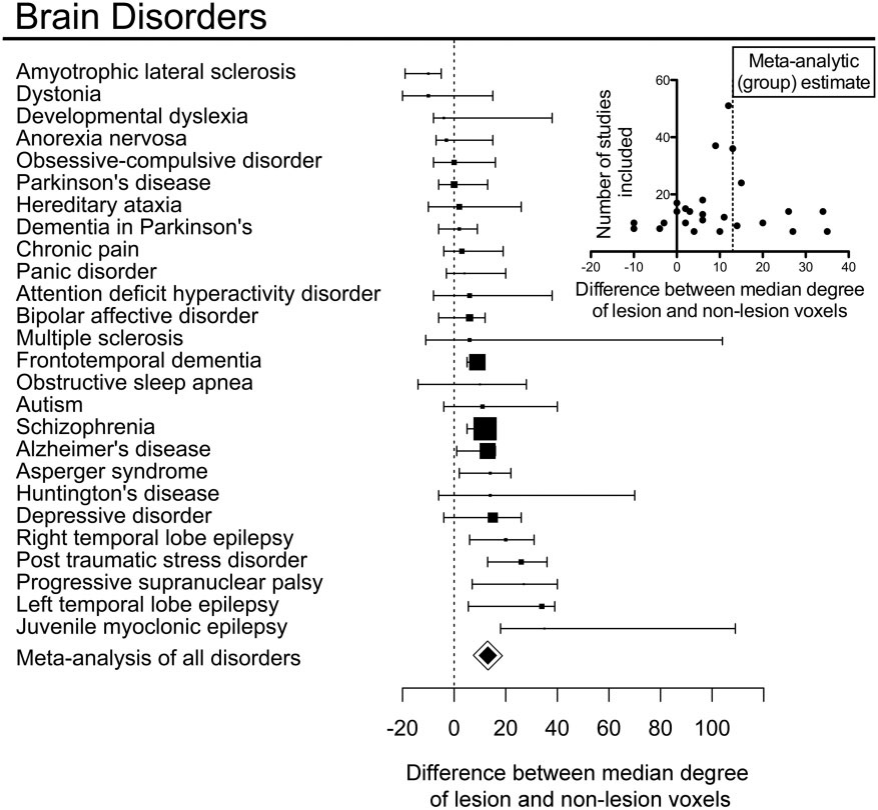
Hub concentration of lesions is common to many specific brain disorders. For each of 26 disorders, box plots represent the difference in median degree of lesion voxels versus non-lesion voxels with a bootstrap 95% CI; the size of the box is proportional to the number of primary studies in the MRI literature. Small inset plot shows the relationship between sample size and difference in median degree for every study. Note that results from individual disorders are symmetrically distributed around the meta-analytical summary of all disorders, with a larger variance observed for disorders represented by fewer studies.

We investigated the relationship between normative hubs and MRI lesions defined by specific meta-analyses of the primary VBM literature for each of the 26 disorders (Fig. 6). The median degree of lesion voxels was greater than non-lesion voxels in 20 of 26 disorders; in four disorders, the median degree was reduced in lesion voxels; and in two disorders there was no difference. Plotting the effect sizes against the number of studies used in each meta-analysis (a so-called funnel plot) showed a symmetrical distribution centred around the effect size from the meta-analysis pooling all disorders together, suggesting that the variability of estimated effect sizes across individual disorders might be related to the variable number of primary studies reported for each disorder (Fig. 6 inset). Bootstrap confidence intervals for the effect size of each specific disorder indicated that lesion voxels had a significantly higher degree than non-lesion voxels in nine disorders (Fig. 6): Alzheimer’s dementia, Asperger’s syndrome, frontotemporal dementia, juvenile myoclonic epilepsy, left and right temporal lobe epilepsy, progressive supranuclear palsy, post-traumatic stress disorder and schizophrenia. In only one disorder, amyotrophic lateral sclerosis, was the degree of lesion voxels significantly lower than the degree of non-lesion voxels.

**Figure 7:**
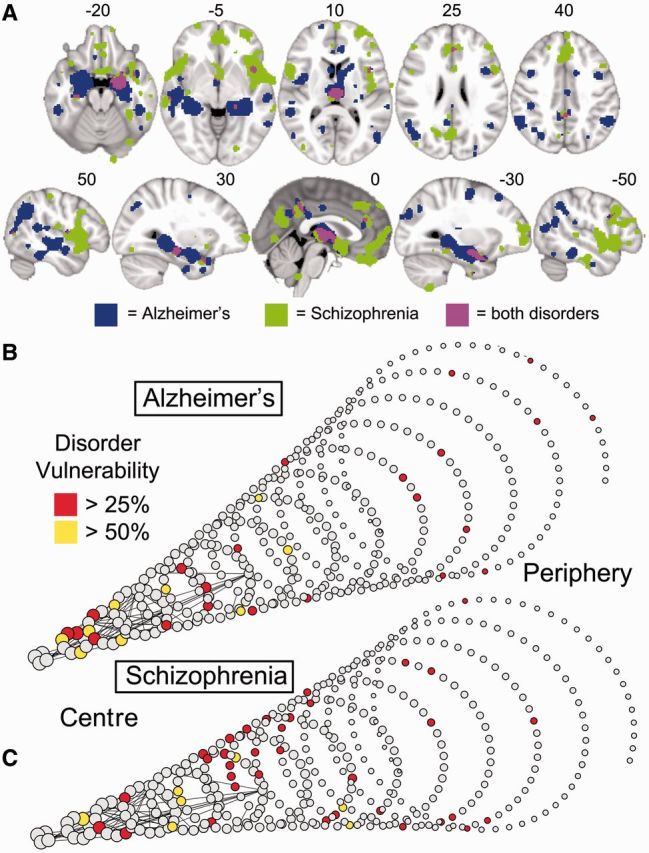
Schizophrenia and Alzheimer’s disease impact mainly on anatomically distinct subsets of hubs. (**A**) Meta-analytic maps of cortical and subcortical lesions associated with schizophrenia (green voxels), or Alzheimer’s disease (blue voxels), or both disorders (pink voxels). (**B** and **C**) Lesions mapped in spiral networks in schizophrenia (**B**) and Alzheimer’s disease (**C**) where the tip represents the highest degree nodes for both disorders and the strongest 0.1% of edges are shown.

Although lesions were concentrated on hubs in most disorders, the anatomical identity of the lesioned hubs was different between different disorders. This was illustrated by the examples of Alzheimer’s disease and schizophrenia, the two disorders most frequently studied in the primary literature. In both disorders, high degree nodes were significantly more likely to be lesioned (Figs 6, 7B and C), but the anatomical location of lesions was largely disorder-specific: medial frontal and anterior cingulate regions were most affected in schizophrenia, whereas medial temporal and parietal regions were most affected in Alzheimer’s disease (Fig. 7A). Only the thalamus and hippocampus were consistently lesioned in both. The difference between disorders was also highlighted by analysis of the relationship between lesion concentration and degree within specific cortical lobes and subcortical structures (Fig. 5B and C). In Alzheimer’s disease, the relationship between lesions and degree was strongest within temporal regions (2.6% increase in the probability of a lesion per degree; *P < *10^−4^, permutation test); whereas in schizophrenia, it was present within frontal, temporal, and parietal lobes (increases of 2.6%, 1.1% and 2.2%, respectively; all *P < *0.01, permutation tests).

### Generalization to a functional connectome

One potential limitation of these results is that they are contingent on a network model based on a single modality of neuroimaging (DTI) data on one sample of healthy volunteers (*n* = 56). We therefore conducted a parallel analysis based on a different normative human brain network model, estimated meta-analytically from a large sample of previously published functional neuroimaging data (Crossley *et al.*, 2013). This functional coactivation network shared many topological properties with the DTI network, including the existence of hubs, modules, small-worldness and rich clubs (Supplementary Fig. 3). Resilience analysis of the functional coactivation network demonstrated that its global efficiency was vulnerable to targeted attack on hubs (Supplementary Fig. 4). Pathological lesions defined by the meta-analysis of all disorders were concentrated on hubs of the functional coactivation network. The median weighted degree of lesion voxels was 3.38, compared to 2.77 in non-lesion voxels; *P < *10^−3^, permutation test. Likewise, logistic regression analysis estimated a 17.3% increase in the probability of a voxel being lesioned for unit increase in the weighted degree of the corresponding node (*P < *10^−4^, permutation test, Fig. 3E). Rich-club nodes of the functional co-activation network were also almost twice as frequently lesioned as peripheral nodes (19.8% of lesion voxels in rich-club nodes, 10.3% in peripheral nodes; *P < *0.013, permutation test). There was a positive trend for lesion probability to increase as a logistic function of nodal degree in all anatomical subsets (basal ganglia; frontal, parietal, temporal and occipital cortices; Supplementary Fig. 5) although this was statistically significant only for frontal regions (22% increase of lesion probability per unit increase in weighted degree; *P* < 10^−4^, permutation test). Lesion probability was also significantly related to nodal degree in all cortical regions, excluding basal ganglia (16.5% increase, *P* < 10^−4^, permutation test).

### Methodological variations

To further test the robustness of the results of analysis of the DTI connectome, we explored a number of reasonable variations in the methodology used for network analysis and for meta-analytic lesion mapping. Results were substantively unchanged by variation in the methods used to construct the normative DTI connectome, such as excluding basal ganglia (Hagmann *et al.*, 2008), or building a weighted network where the strength of each edge was weighted by the number of tractographic streamlines connecting the two nodes (Supplementary Fig. 6A). The relationship between anatomical lesions and hubs was reproduced when alternative plausible metrics of topological centrality, including participation coefficient (Meunier *et al.*, 2009, 2010; Power *et al.*, 2013), distance-weighted degree (Liu *et al.*, 2014*b*) and betweenness centrality (Rubinov and Sporns, 2010), were used to define hubs (Supplementary Fig. 6B). Likewise, modifying aspects of the analysis of anatomical lesions did not substantially change the main findings. Results were robust to a leave-one-out disorder analysis, excluding neurodegenerative disorders, or including fewer disorders represented by a larger number of primary studies (Supplementary Fig. 6C).

## Discussion

Overall, these results provide evidence in support of the theoretical prediction that brain hubs are central to brain disorders in general. Computational analysis confirmed and extended previous *in silico* brain studies (Achard *et al.*, 2006; Kaiser *et al.*, 2007; Iturria-Medina *et al.*, 2008; Alstott *et al.*, 2009), demonstrating that targeted attacks on hubs, or the longer distance edges, of the normal DTI connectome were especially likely to degrade its global efficiency. Meta-analysis of a large database of clinical MRI studies demonstrated that hub nodes of the DTI connectome were more likely to be pathologically lesioned by a wide range of brain disorders, results that were reproduced in relation to an independent normal functional brain network. These results based on meta-analysis of case-control VBM studies are in line with recent graph theoretical analyses of resting state functional MRI or DTI data that demonstrated abnormalities of hubs in several disorders including, for example, schizophrenia (Rubinov and Bullmore, 2013), Alzheimer’s disease (Buckner *et al.*, 2009), frontotemporal dementia (Agosta *et al.*, 2013), Parkinson’s disease (Baggio *et al.*, 2014), temporal lobe epilepsy (Liu *et al.*, 2014*a*), Gilles de la Tourette syndrome (Worbe *et al.*, 2012), acute brain injury (Achard *et al.*, 2012), and migraine (Liu *et al.*, 2012).

One immediately interesting observation is that, in the pooled meta-analysis of MRI lesions across all disorders, a certain subset of brain regions was more likely to be affected ‘on average’ across a range of pathogenic processes. One might have reasoned that since each disorder has a specific pathogenesis, it might be expected to affect a specific set of anatomical regions, and therefore the average over disorders would demonstrate no consistently abnormal lesions. In contrast, the experimental observation is more compatible with previous suggestions (Saxena and Caroni, 2011; Jagust, 2013; Menon, 2013) that an anatomically defined subset of brain regions are generally more implicated in brain disorders; in other words, that some brain regions are relative hotspots for clinical abnormality of grey matter volume. The main value added by our topological analysis is the evidence we provide that these disorder-general lesions are concentrated in hubs of the normal connectome.

### Hubs and lesions

Network science has provided insights into general principles governing the interaction of multiple elements (or nodes) of a complex system, whether these nodes represent people, computers or proteins. Topological analysis of complex networks has previously demonstrated that scale-free networks, with fat-tailed degree distributions indicating the existence of hubs, are vulnerable to attack on hub nodes (Albert *et al.*, 2000). For example, hub proteins, as defined by the scale-free network of interactions between proteins, are preferentially targeted by pathogens in plants (Mukhtar *et al.*, 2011) and humans (Calderwood *et al.*, 2007), and their mutations are lethal in yeast (Jeong *et al.*, 2001). Echoing these results, here we provide evidence that the hubs of brain networks are generally central to clinical disorders.

Our primary measure of ‘hubness’ has been degree centrality, meaning that brain regional nodes with the highest number of connections (edges) to other regions are the most frequently lesioned by diseases. Such high degree nodes are often connector hubs, connecting to regions in different brain modules. As a secondary criterion of hubness, we used participation coefficient, which measures the proportion of intermodular connections for each node (Guimera and Nunes Amaral, 2005), to show that the brain regions most critical to intermodular communication were also more likely to be pathologically lesioned. A third measure of centrality, related to both nodal degree and participation coefficient, is the elite clique of highly interconnected hub nodes that exists in networks with a high rich-club coefficient (van den Heuvel and Sporns, 2011). We showed that lesions were also disproportionately concentrated in rich-club regions rather than in the much larger number of peripheral regions.

Why should lesions be concentrated in hubs? We suggest that there are at least two major convergent factors. First, hubs are more functionally valuable, especially for ‘higher-order’ cognitive tasks and adaptive behaviour; therefore lesioned hubs are more likely to be symptomatic than lesioned non-hubs. Second, hubs are more biologically costly and therefore more vulnerable to a diverse range of pathogenic processes.

### Hub pathology: high value/high cost brain regions are more frequently lesioned by disorders

From the perspective of brain and cognition, studies have shown that higher global efficiency of functional brain networks (derived from resting-state functional MRI data) is positively correlated with better cognitive performance (van den Heuvel *et al.*, 2009; Giessing *et al.*, 2013); and that cognitively more demanding tasks lead to the appearance of long-range integrative connections in task-related MEG data (Kitzbichler *et al.*, 2011). These data are broadly aligned with workspace theories that ‘higher order’ cognitive functions depend on a more integrative network topology (Dehaene and Changeux, 2011). In particular, the functional connectome used to define hubs in this analysis has previously been reported to include a rich club of highly-interconnected hub nodes that were coactivated by a variety of tasks, especially executive tasks demanding both cognition and action, highlighting the topological value of hubs (and clubs) for integrative processes and adaptive behaviour in healthy humans (Crossley *et al.*, 2013). On this basis, it seems intuitive that pathological lesions to network hubs are especially likely to be associated with clinically significant cognitive impairments.

Hubs of brain networks are also arguably biologically expensive. Biological cost of brain networks can be measured in many ways, including the local blood flow or metabolic rate of a regional node, and the physical distance of an edge or connection between nodes (the metabolic cost of connections is likely to increase monotonically as a function of distance). Recent studies have indeed shown that high degree and highly efficient hub nodes of normal functional MRI connectomes have higher blood flow, glucose metabolic rates, and longer connection distance, than non-hub nodes (van den Heuvel and Sporns, 2011; Alexander-Bloch *et al.*, 2013; Liang *et al.*, 2013; Tomasi *et al.*, 2013).

The extra biological cost of hubs may be a price worth paying for their topological value, but it may also increase their vulnerability to diverse disease processes. For example, any disease process that restricts neuronal metabolism, e.g. ischaemia or oxidative stress, might be expected to have disproportionate impact on the most metabolically active (hub) nodes. Likewise any disease process that damages long-distance axonal projections (e.g. disseminated sclerosis or traumatic brain injury), or disrupts active axonal transport mechanisms, might be expected to disconnect hubs and/or to disrupt long-range communication between network modules.

However, we note that lesions might be concentrated on hubs purely because of their greater topological value: some disease processes might affect brain regions with uniform probability but lead to symptoms when the lesion happened to be in a hub; or some disease processes might be initiated locally, perhaps in a peripheral node, and only become symptomatic once they have propagated to topologically central nodes. In other words, although it seems plausible that hubs are not just more symptomatic but also more vulnerable, the claim about vulnerability is less securely established. More direct evidence for hub vulnerability is required; this might include experiments in which a global (uniform probability) insult is administered in animal models. The hub vulnerability hypothesis would predict that an acute severe global insult would cause greater structural and functional damage to hub regions than to non-hubs. Experimental lesions in animals caused by global hypoxia, mitochondrial dysfunction (Melov, 2004), or diffuse axonal injury as seen in models of traumatic brain injury (Xiong *et al.*, 2013) or multiple sclerosis (Ransohoff, 2012), could be revisited within this framework.

Although we claim that the anatomical lesions of brain disorders are generally concentrated on normal network hubs, this does not mean that different disorders will necessarily involve an identical set of hubs, as shown for Alzheimer’s disease and schizophrenia. This presumably reflects important differences in their respective pathogenetic processes. As such, we do not suggest that topological centrality is the only factor determining the location of lesions in different disorders. Lesions in specific disorders such as Alzheimer’s disease and schizophrenia are located in high degree regions, but not necessarily in the highest degree hubs, and not hub-concentrated to the same extent within different lobes of the cortex. Disorder-specific factors will presumably determine which brain regions are affected first and how different neurodevelopmental and neurodegenerative disease processes then propagate over the network architecture.

### Robustness and generalizability of results

We have used meta-analytic methods to define structural MRI lesions and functional network hubs on the basis of large numbers of primary data published by research groups worldwide. Thus many of these results are based on a large proportion of the extant relevant scientific literature.

The DTI connectome was estimated on the basis of a smaller data set but we showed that its properties were consistent with previously published DTI connectomes and were robust to variation in the methods used to construct the connectome. The basic association between lesion probability and nodal degree demonstrated for the DTI connectome globally was also evident for frontal and temporal cortical subsets of the connectome, indicating that both cortical and subcortical hubs are implicated in disorders.

Although DTI provides the most direct access to the structural network of the living human brain, it also has limitations (Morris *et al.*, 2008; Jones and Cercignani, 2010; Craddock *et al.*, 2013; Jones *et al.*, 2013). By using a completely different approach to build the connectome, based on meta-analysis of task-based functional neuroimaging studies, we were able to replicate all the key results linking MRI lesions to network hubs. This functional co-activation network was based on a different parcellation template, further suggesting that our results are not driven by the specific parcellation scheme used for the DTI analysis.

We also tested the extent to which our results were likely to be driven by a few disorders. Our meta-analytic pooling of all disorders balanced the weight each disorder contributed to the general estimate, and we also showed that results were consistent across several subgroups of disorders, e.g. after excluding all neurodegenerative disorders. However, there was not statistically robust evidence for hub-targeting lesions in all of the 26 disorders we evaluated specifically. This could mean that some disorders (such as amyotrophic lateral sclerosis) may truly not be hub-concentrated. One could argue that a substantial proportion of patients with amyotrophic lateral sclerosis have only motor symptoms (Goldstein and Abrahams, 2013), and hypothesize that the association between hubs and lesions may be most evident for disorders associated with clinical abnormalities of integrative function such as cognitive impairment or seizures. However, many of the disorders for which the evidence for preferential lesioning of hubs was weaker had been studied in a relatively small number of patients, suggesting that there may also have been insufficient power to link the anatomical distribution of lesions to the underlying network topology in some of these less frequently studied disorders.

## Conclusion

High cost/high value hubs in normal human brain networks are more likely to be anatomically abnormal than non-hubs in many (if not all) brain disorders.

## Supplementary Material

Supplementary Fig. 1

## Abbreviations

DTI: diffusion tensor imaging
VBM: voxel-based morphometry
