# Supplementary material for: The hubs of the human connectome are generally implicated in the anatomy of brain disorders
Source: Brain. 2014 Jun 19;137(8):2382–95. doi: 10.1093/brain/awu132 (PMC4107735; doi:10.1093/brain/awu132)
Supplement: Supplementary Fig. 1 [file brain_awu132_index.html]

Supplementary Data | Brain

## Supplementary Data

files

**Files in this Data Supplement:**

- Supplementary Data - docx file
- Supplementary Data - docx file
