# Supplementary material for: The hubs of the human connectome are generally implicated in the anatomy of brain disorders
Source: Brain. 2014 Jun 19;137(8):2382–95. doi: 10.1093/brain/awu132 (PMC4107735; doi:10.1093/brain/awu132)
Supplement: Supplementary Fig. 1 [file f6f94ae717c4a2e9c78e26131eb20fa4_brain-2013-01933-File009.docx]

**The hubs of the human connectome are generally implicated in the anatomy of brain disorders**

Nicolas A. Crossley, Andrea Mechelli, Jessica Scott, Francesco Carletti, Peter T. Fox, Philip McGuire and Edward T. Bullmore.

**Supplementary Information**

**Supplementary Methods**

- Graph-theoretical metrics used in this study.

- Accessing the VBM data from BrainMap.org.

- Balancing sample sizes of studies in different disorders.

**Supplementary Figures**

- **Figure S1.** Size of the regions in the DTI template and their relationship to degree and probability of a "lesion" voxel.

- **Figure S2.** Flow diagram detailing the strategy used in the literature search and the extraction of data from primary studies.

- **Figure S3.** Topological characteristics of the normal brain functional network (coactivation network).

- **Figure S4.** Computational attacks and the resilience of the functional coactivation network.

- **Figure S5.** Degree and probability of lesion in anatomical subnetworks in the coactivation network template.

- **Figure S6.** Robustness analyses.

**Supplementary Tables**

**Table S1**. Stereotactic coordinates of disorder-general grey matter abnormalities identified by meta-analysis of voxel-based morphometry studies of 26 clinical brain disorders.

**Supplementary Methods**

**Graph-theoretical metrics used in this study**

Several graph-analytic metrics were derived from the DTI and functional coactivation networks. We detail them below:

- ***Degree***: Number of significant connections that a node has.

- ***Weighted degree*** *(strength):* Nodal characteristic which describes the sum of all the weights of its connections.

- ***Shortest path***: The shortest topological distance (geodesic) between two nodes. The shortest path of a network is the average of all the shortest paths between all pairs of nodes. λ is the ratio between the shortest path of the network and comparable random networks (same number of nodes and edges, same degree and weight distribution) ([Watts and Strogatz, 1998](#_ENREF_11)).

- ***Clustering***: The proportion of existing links between the neighbours of a node from the possible ones, where neighbours of a node are the nodes directly connected to them. γ is the ratio between the clustering of the network analysed and the clustering of comparable random networks (same number of nodes and edges, same degree and weight distribution) ([Watts and Strogatz, 1998](#_ENREF_11)).

- ***Global efficiency***: The average of the inverse of the shortest paths between all nodes. It can be used in weighted or disconnected networks ([Latora and Marchiori, 2001](#_ENREF_7)).

- ***Small-world*** (σ): This is the ratio between γ and λ ([Humphries and Gurney, 2008](#_ENREF_5)).

- ***Modularity***: The best partition of the network into subgroups which are highly connected between themselves. This is obtained by maximising a parameter *Q* ([Newman, 2006](#_ENREF_8)):

$$Q=\frac{1}{4m}\sum_{ij} \left( A_{ij}-\frac{k_{i}k_{j}}{2m} \right)s_{i}s_{j}$$

where *A_ij_* is the edge between nodes *i* and *j*, with degrees denoted *k_i_* and *k_j_*, *m* denotes the total number of edges of the network, and *s_i_s_j_* is 1 if the nodes belong to the same group or -1 if not.

- ***Participation coefficient*:** Measures how well a node in a given module is connected to other modules, defined as:

$$P_{i}=1-\sum\left( \frac{k_{is}}{k_{i}} \right)^{2}$$

where *k_is_* is the sum of the connections from node *i* to module *s*, and *k_i_* its degree. As such, nodes with links uniformly distributed to all modules will have a coefficient near one, and if only connects other nodes within its modules, near zero ([Guimera and Nunes Amaral, 2005](#_ENREF_4)).

- ***Rich-club coefficient***: Describes whether high degree nodes in a network tend to be highly connected between themselves. We here used the version for binary networks (DTI network) and for weighted networks (coactivation network):

- *for binary networks*: describes the number of connections between high degree nodes comparing them to the possible number of connections:

$$\varphi\left( k \right)=\frac{2E_{>k}}{N_{k}\left( N_{k}-1 \right)}$$

where *N_k_* are the nodes that have a degree higher than a given value *k*, and *E_k_* are the number of edges among the *N_k_* nodes ([Colizza *et al.*, 2006](#_ENREF_1)).

\ - *for weighted networks*: is the proportion of the strongest edges of the network that connect high degree nodes. Formally, this is defined for a cut-off richness factor *r* as:

$$\varphi^{w}=\frac{W_{>r}}{\sum_{l=1}^{E>r} w_{l}^{rank}}$$

where *W_>r_* is the sum of the weights of the connections between nodes with a richness factor higher than *r*, *E>r* the number of these edges, and the denominator describes the sum of the top *E>r* strongest edges of the network. We here used weighted degree as the richness factor to rank the nodes ([Opsahl *et al.*, 2008](#_ENREF_9)).

Both rich-club coefficients for binary and weighted networks can be normalised by the same parameter obtained from a null model of random networks with the same degree (and weight) distribution.

**Accessing the VBM data from BrainMap.org.**

As described in the main text and shown in figure S1, electronic searches for each individual disorder were performed in Pubmed. Any identified VBM study not included at the time of the search in the BrainMap database ([Fox and Lancaster, 2002](#_ENREF_3); [Fox *et al.*, 2005](#_ENREF_2); [Laird *et al.*, 2005](#_ENREF_6)) were subsequently added. As such, all VBM data used in this study is available for public access.

Readers can peruse the list of VBM studies in the supplementary file included, and access details of the specific studies they wanted from the BrainMap database. Otherwise, they might want to download the Sleuth workspaces for each of the 26 included disorders from BrainMap.org/pubs. Workspaces consist of details of the included studies, the specific experiments (or contrasts), and the possibility of plotting the reported coordinates in a brain map. Note that workspaces only contain the studies reporting significant decreases in grey matter volume in patients. We have also included studies reporting no differences when calculating the confidence intervals of the individual meta-analysis using bootstrap (**Fig. 6** of the main text).

**Balancing sample sizes of studies in different disorders**

As described in the text, we performed an ALE meta-analyses pooling studies from the 26 disorders included. In order to avoid certain disorders dominating the results, we first included the same number of studies per disorder (7). Although this partly balances the weight each disorder has on the summary measure, it does not control for certain disorders (particularly those with a higher number of published studies) having studies with larger samples. We therefore calculated the precision of each study included in this analysis using a sample size (defined here as "sample size for balancing purposes" or "*bSS*") defined by the following formula:

$${bSS}_{study n}= {oSS}_{study n} \times\frac{{pSS}_{disorder of study n}}{min (pSS)}$$

where *oSS* is the original sample size of the study, and *pSS* the pooled sample size for each disorder (sum of the subjects included in the seven studies for that disorder). This correction increases the uncertainty of the differences modeled using the peak coordinates of larger studies, avoiding them to dominate the overall summary estimate. Of note, the relationship of studies within disorders is maintained, so that larger studies still provide a more reliable estimate of the differences for that disorder than the smaller studies.

**Figure S1**

**
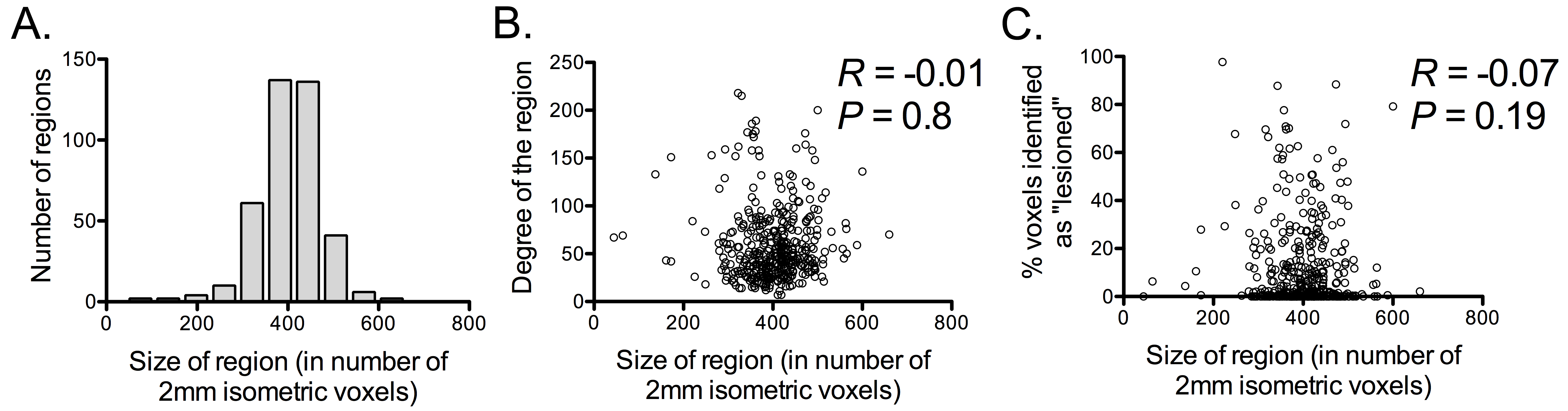
**

**Figure S1. Size of the regions in the DTI template and their relationship to degree and probability of a "lesion" voxel.** A) Histogram showing small dispersion around mean size of nodes (400 voxels = approx. 3.2 cm^3^; standard deviation = 71 voxels or 0.57 cm^3^; coefficient of variation (100 x SD/mean) = 17.8%). B) Relationship between nodal volume and degree, showing that the small variability of nodal volume has no effect on the degree. C) Similarly, there is no relationship between nodal volume and the probability of having a "lesion" voxel. This analysis was performed on the DTI template, but the alternative coactivation template is based on similar principles.

**Figure S2.
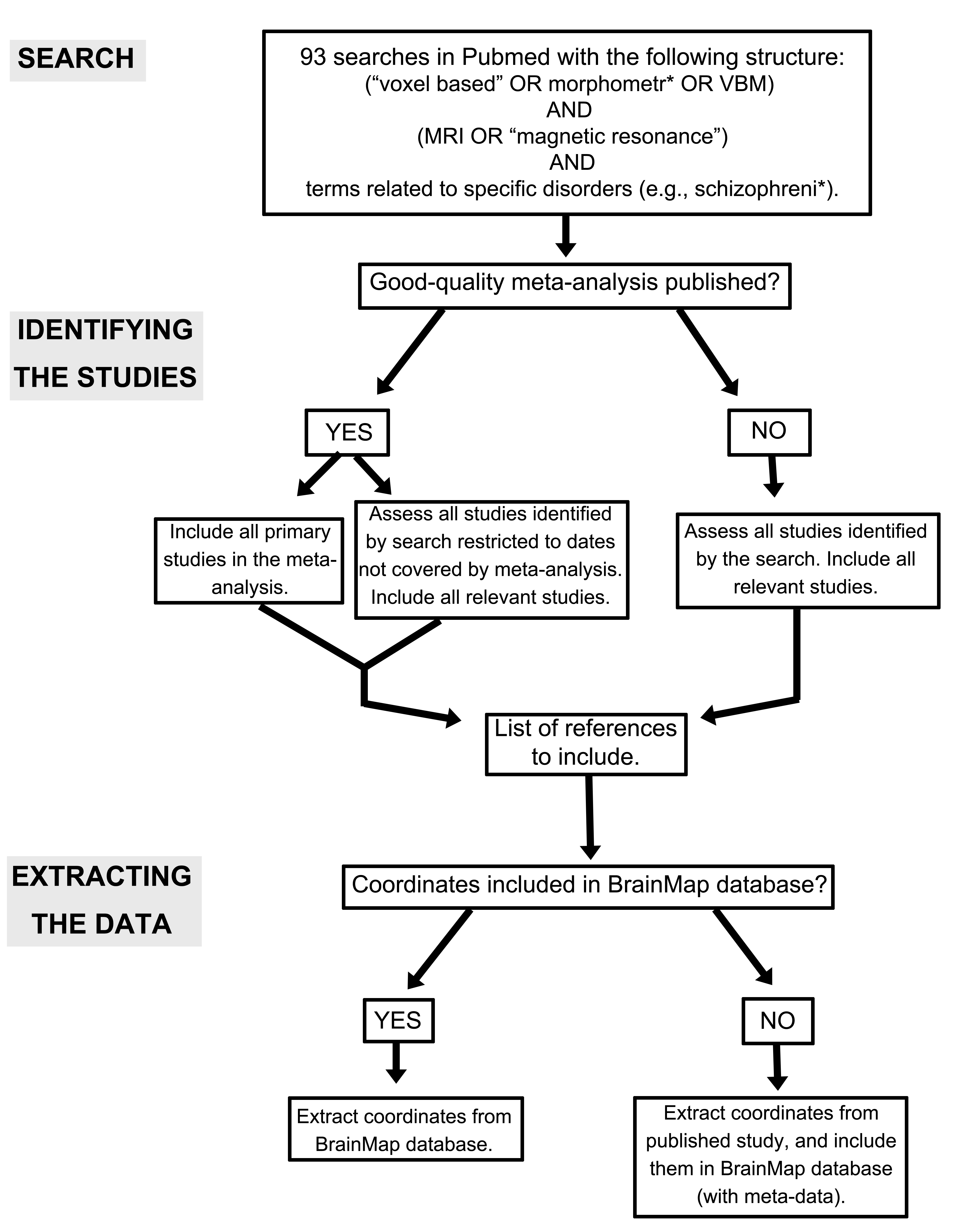
**

**Figure S2. Flow diagram detailing the strategy used in the literature search and the extraction of data from primary studies.**

**Figure S3**


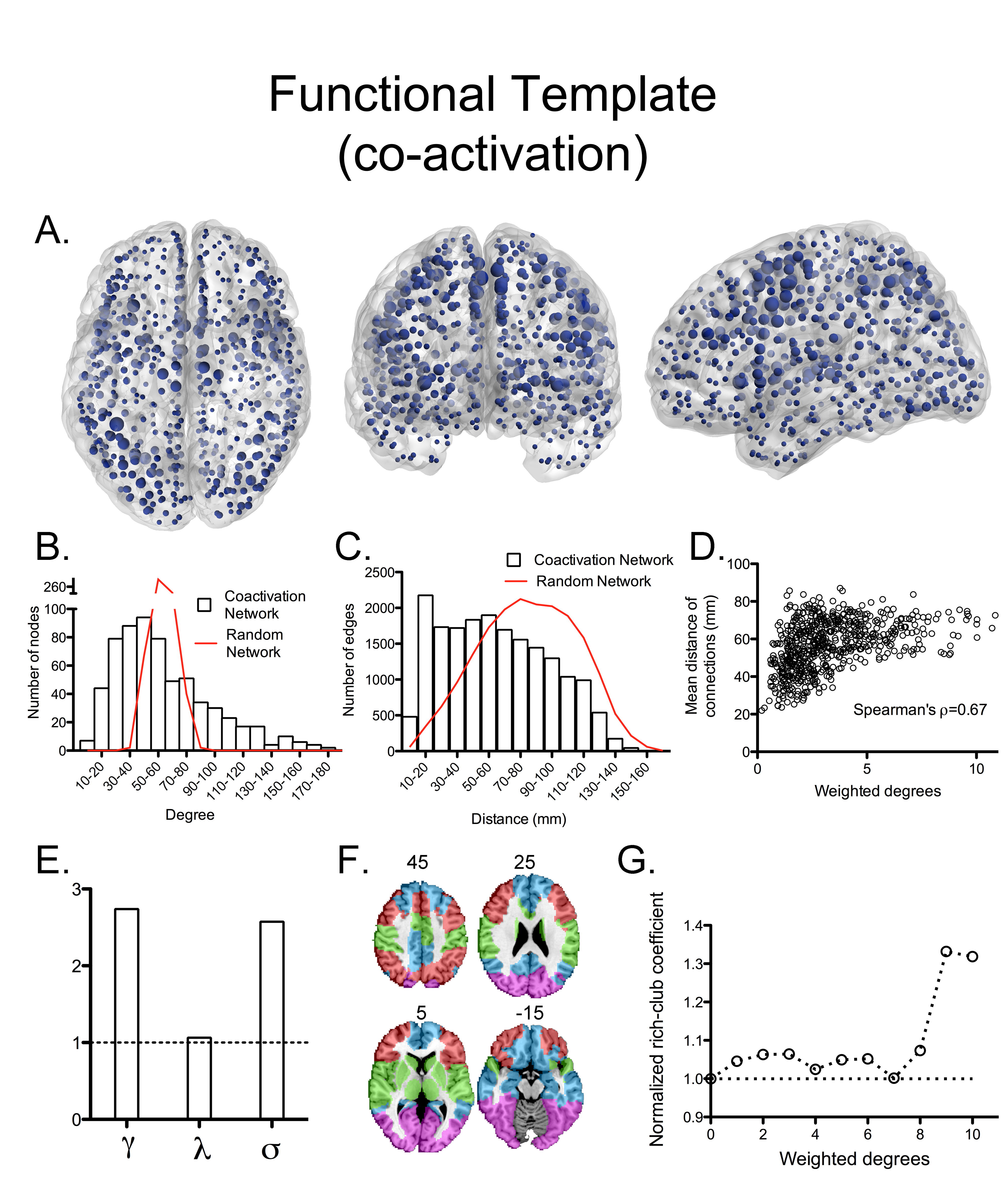


**Figure S3. Topological characteristics** **of the normal brain functional network (coactivation network).** A) Nodes of the network in anatomical space; the size of each node is proportional to its weighted degree. B) Fat-tailed degree distribution of the coactivation network (histogram) indicating higher probability of hubs than in a random (Erdös-Rényi) graph (red line). C) Distance distribution of coactivation network (histogram) and of random graphs matched for degree distribution (red line). It should be noted that degree in this network was positively correlated to participation coefficient (*R*=0.15). D) Scatterplot of weighted degree *versus* mean connection distance in the coactivation network. E) Small-world properties of the network (γ = normalised clustering coefficient; λ = normalised path length; σ = ratio of γ to λ; dotted line = 1, the expected value of all these metrics in a random graph). F) Modular decomposition of the coactivation network. As a non-correlation network, the size of its modules explained a comparatively low proportion of the variance (9%)([Power *et al.*, 2013](#_ENREF_10)). G) Plot of the normalised rich club coefficient (*y*-axis) as a function of weighted degree threshold (*x*-axis) used to define the rich club; dotted line = 1, the expected value of the normalised rich club coefficient in a random network with the same degree and weight distribution as the coactivation network.

**Figure S4.**


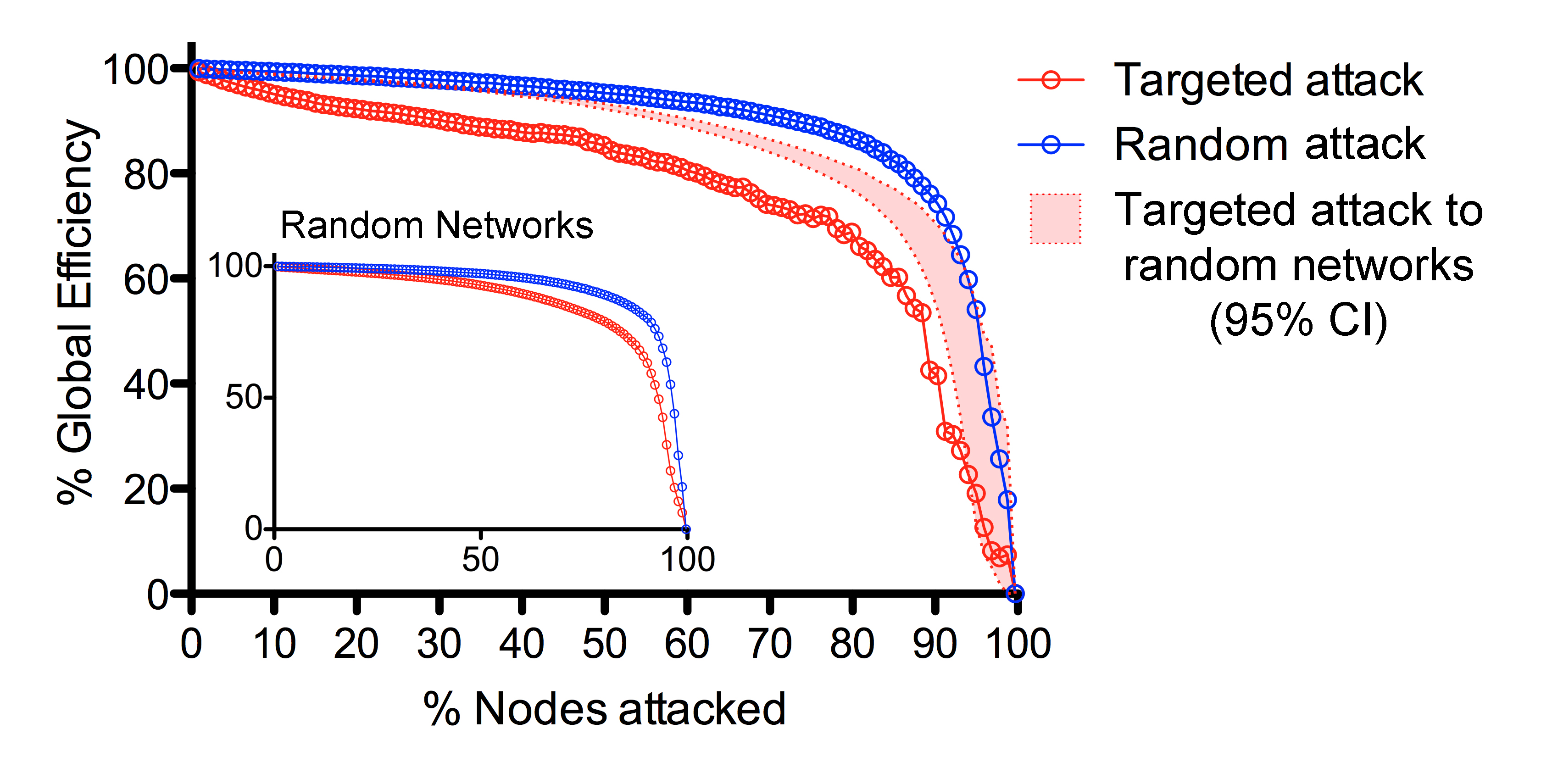


**Figure S4. Computational attacks and the resilience of the functional coactivation network.** Plot of global efficiency of the coactivation network *versus* percentage of nodes deleted. When nodes are deleted randomly the efficiency of the network is approximately as resilient as a random (Erdös-Rényi) graph (inset and red area in main graph); when high degree nodes are targeted (deleted in order of decreasing degree) the efficiency of the network degrades more rapidly than a random graph.

**Figure S5**

**
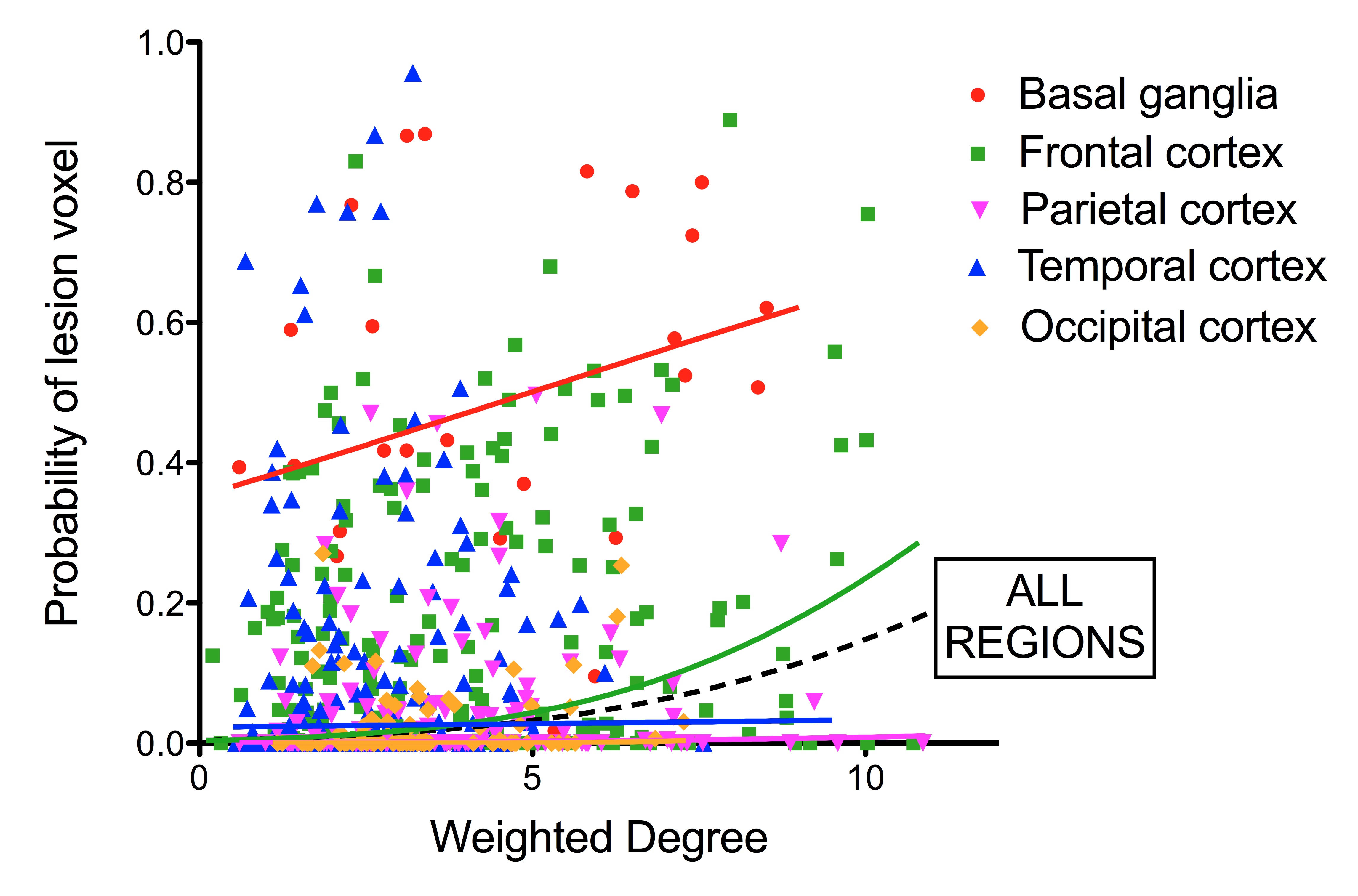
**

**Figure S5. Degree and probability of lesion in anatomical subnetworks in the coactivation network template.** Probability of lesion in a voxel and degree of each of the 638 regions of the coactivation network. Nodes have been colour-coded according to their anatomical (lobar) location. Logistic regression lines for each subgroup and for all the regions are shown.

**Figure S6**

**
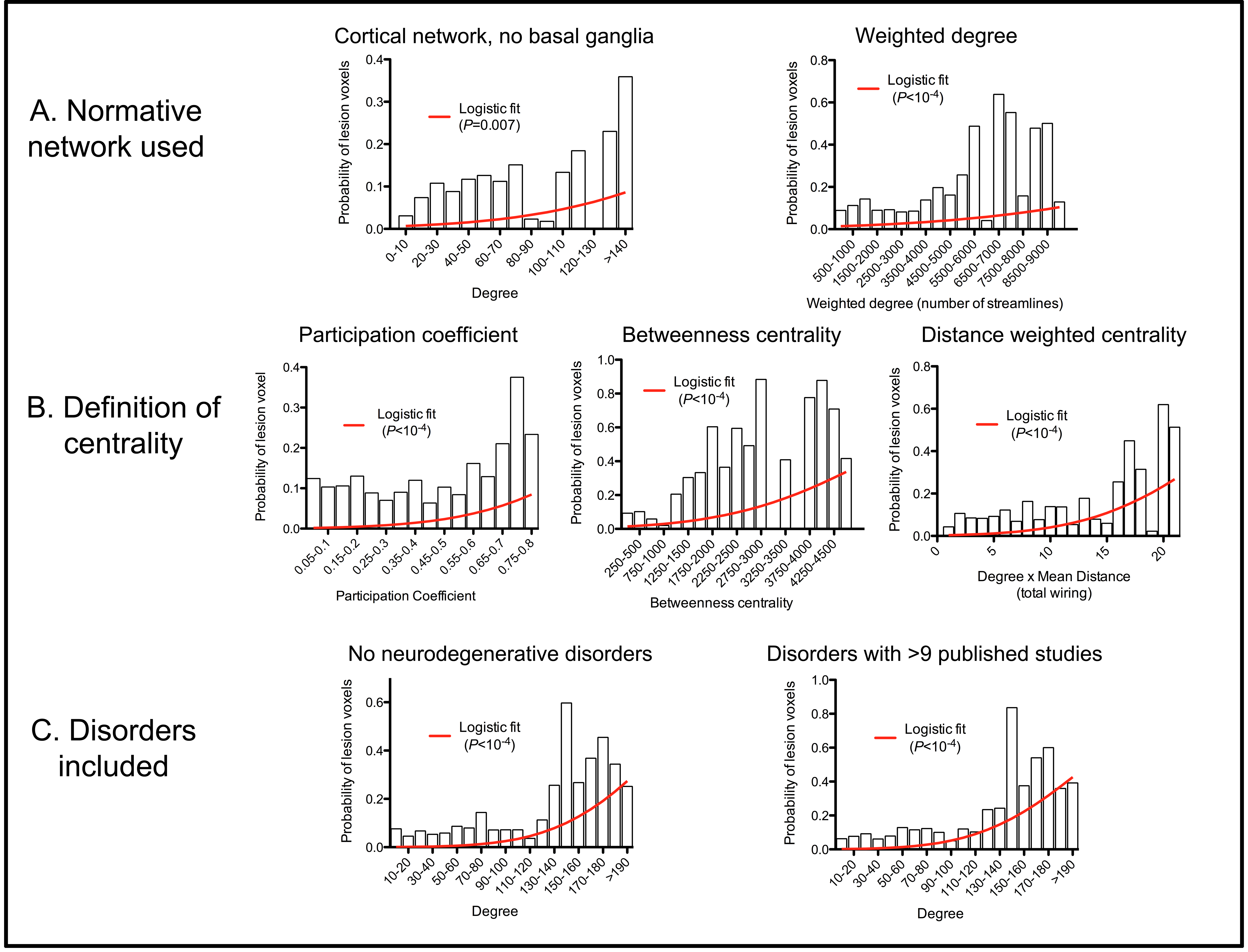
**

**Fig S6. Robustness analyses.** Several analyses were performed using different versions of the DTI connectome (A), different measures of nodal centrality (B), and different meta-analytic lesion maps (C). In each case, the probability of lesion voxels (y-axis) was significantly associated with nodal centrality (x-axis) as highlighted by fitted logistic regression curves (red lines).

**Supplementary Table**

**Table S1. Stereotactic coordinates of disorder-general grey matter abnormalities identified by meta-analysis of voxel-based morphometry studies of 26 clinical brain disorders**

| **MNI COORDINATES** | | | **PEAK VALUE OF META-ANALYTIC (ALE) SUMMARY** | **ANATOMICAL REGION** |
| --- | --- | --- | --- | --- |
| **x** | **y** | **z** |  |  |
| 10 | 4 | 8 | 0.0508 | Right caudate body |
| 2 | -16 | 10 | 0.0507 | Right thalamus |
| -30 | -16 | -18 | 0.0475 | Left hippocampus |
| -24 | -6 | -20 | 0.0454 | Left amygdala |
| 42 | 18 | 4 | 0.0402 | Right insula, BA 13 |
| -28 | -38 | -2 | 0.0400 | Left hippocampus |
| -10 | -28 | 8 | 0.0398 | Left thalamus, pulvinar |
| -2 | -2 | -14 | 0.0398 | Hypothalamus |
| 28 | -4 | -18 | 0.0395 | Right amygdala |
| 34 | -18 | -16 | 0.0390 | Right hippocampus |
| -12 | 12 | 8 | 0.0378 | Left caudate, body |
| -38 | 22 | 0 | 0.0370 | Left insula, BA 13 |
| 44 | -10 | 8 | 0.0367 | Right insula, BA 13 |
| 10 | 16 | 0 | 0.0360 | Right caudate, head |
| 46 | -16 | 14 | 0.0336 | Right insula, BA 13 |
| -24 | 2 | 10 | 0.0335 | Left putamen |
| -54 | -14 | 42 | 0.0333 | Left postcentral gyrus, BA 3 |
| -4 | 8 | 0 | 0.0327 | Left caudate, head |
| 50 | 12 | 24 | 0.0322 | Right inferior frontal gyrus, BA 9 |
| 52 | -10 | 38 | 0.0317 | Right precentral gyrus, BA 4 |
| 12 | -32 | 2 | 0.0317 | Right thalamus, pulvinar |
| -46 | 12 | 32 | 0.0317 | Left inferior frontal gyrus, BA 9 |
| -52 | 20 | 16 | 0.0309 | Left inferior frontal gyrus, BA 45 |
| -56 | -4 | 2 | 0.0295 | Left superior temporal gyrus, BA 22 |
| 18 | -14 | 6 | 0.0293 | Right thalamus, ventral posterior lateral nucleus |
| -42 | 54 | -10 | 0.0286 | Left inferior frontal gyrus, BA 10 |
| -28 | 8 | -24 | 0.0282 | Left inferior frontal gyrus, BA 47 |
| 52 | 6 | -22 | 0.0278 | Right middle temporal gyrus, BA 38 |
| -50 | 32 | 22 | 0.0260 | Left middle frontal gyrus, BA 46 |
| 56 | -20 | 46 | 0.0242 | Right postcentral gyrus, BA 2 |
| 44 | 30 | 34 | 0.0241 | Right precentral gyrus, BA 9 |
| -46 | 10 | 48 | 0.0240 | Left middle frontal gyrus, BA 6 |
| 24 | 4 | 8 | 0.0240 | Right putamen |
| 34 | 12 | -22 | 0.0238 | Right inferior frontal gyrus, BA 47 |
| 4 | 16 | -20 | 0.0237 | Right subgenual area, BA 25 |
| 40 | -26 | 50 | 0.0204 | Right postcentral gyrus, BA 3 |
| -48 | 16 | -18 | 0.0189 | Left superior temporal gyrus, BA 38 |
| 62 | -8 | 0 | 0.0179 | Right superior temporal gyrus, BA 22 |
| 54 | 6 | 6 | 0.0177 | Right precentral gyrus, BA 44 |
| -2 | 46 | 24 | 0.0398 | Left medial fronta, BA 9 |
| 4 | 46 | 14 | 0.0264 | Right anterior cingulate, BA 32 |
| -30 | -18 | 64 | 0.0300 | Left precentral gyrus, BA 4 |
| 6 | -24 | 54 | 0.0300 | Right paracentral lobule, BA 6 |
| -46 | -30 | 46 | 0.0244 | Left inferior parietal lobule, BA 40 |
| -40 | -64 | -12 | 0.0272 | Left fusiform gyrus, BA 37 |
| 54 | -54 | -8 | 0.0235 | Right inferior temporal gyrus, BA 37 |
| -4 | 16 | 28 | 0.0196 | Left cingulate gyrus, BA 24 |
| 4 | -10 | 30 | 0.0248 | Right cingulate gyrus, BA 23 |
| -48 | -68 | 10 | 0.0263 | Left middle temporal gyrus, BA 37 |
| 52 | -64 | 40 | 0.0214 | Right angular gyrus, BA 39 |
| -56 | -52 | 34 | 0.0231 | Left supramarginal gyrus, BA 40 |
| -64 | -32 | 12 | 0.0203 | Left superior temporal gyrus, BA 42 |
| -28 | -76 | 40 | 0.0218 | Left precuneus, BA 19 |
| 2 | -82 | 8 | 0.0205 | Left lingual gyrus, BA 18 |
| 26 | 50 | 20 | 0.0220 | Right superior frontal gyrus, BA 10 |
| -68 | -20 | 6 | 0.0239 | Left superior temporal gyrus, BA 22 |
| -28 | -86 | 18 | 0.0203 | Left middle occipital gyrus, BA 19 |
| -14 | -68 | 22 | 0.0186 | Left precuneus, BA 31 |
| -58 | -48 | 0 | 0.0173 | Left middle temporal gyrus, BA 22 |
| 44 | -76 | 8 | 0.0168 | Right middle occipital gyrus, BA 19 |
| -28 | 44 | 18 | 0.0176 | Left middle frontal gyrus, BA 10 |
| -6 | 48 | -8 | 0.0191 | Left anterior cingulate, BA 32 |
| -48 | -32 | 16 | 0.0174 | Left superior temporal gyrus |
| 20 | -72 | 24 | 0.0179 | Right cuneus, BA 18 |
| -10 | -10 | 48 | 0.0215 | Left paracentral lobule, BA 31 |
| 40 | 46 | 16 | 0.0166 | Right middle frontal gyrus, BA 10 |
| 12 | 62 | 26 | 0.0183 | Right superior frontal gyrus, BA 9 |
| -36 | -62 | 32 | 0.0180 | Left middle temporal gyrus, BA 39 |
| 12 | -50 | 24 | 0.0198 | Right posterior cingulate, BA 30 |
| 24 | 12 | 58 | 0.0163 | Right premotor cortex.BA 6 |
| 46 | -58 | 52 | 0.0188 | Right superior parietal lobule, BA 7 |
| 42 | 10 | -36 | 0.0164 | Right superior temporal gyrus, BA 38 |

BA: Brodmann area.

**References**

Colizza V, Flammini A, Serrano MA, Vespignani A. Detecting rich-club ordering in complex networks. Nat Phys. 2006;2(2):110-5.

Fox PT, Lancaster JL. Opinion: Mapping context and content: the BrainMap model. Nat Rev Neurosci. 2002;3(4):319-21.

Fox PT, Laird AR, Fox SP, Fox PM, Uecker AM, Crank M, et al. BrainMap taxonomy of experimental design: description and evaluation. Hum Brain Mapp. 2005;25(1):185-98.

Guimera R, Nunes Amaral LA. Functional cartography of complex metabolic networks. Nature. 2005;433(7028):895-900.

Humphries MD, Gurney K. Network 'small-world-ness': a quantitative method for determining canonical network equivalence. PLoS One. 2008;3(4):e0002051.

Laird AR, Lancaster JL, Fox PT. BrainMap: the social evolution of a human brain mapping database. Neuroinformatics. 2005;3(1):65-78.

Latora V, Marchiori M. Efficient behavior of small-world networks. Phys Rev Lett. 2001;87(19):198701.

Newman ME. Modularity and community structure in networks. Proc Natl Acad Sci USA. 2006;103(23):8577-82.

Opsahl T, Colizza V, Panzarasa P, Ramasco JJ. Prominence and control: the weighted rich-club effect. Phys Rev Lett. 2008;101(16):168702.

Power JD, Schlaggar BL, Lessov-Schlaggar CN, Petersen SE. Evidence for hubs in human functional brain networks. Neuron. 2013;79(4):798-813.

Watts DJ, Strogatz SH. Collective dynamics of 'small-world' networks. Nature. 1998;393(6684):440-2.
