# Supplementary material for: The hubs of the human connectome are generally implicated in the anatomy of brain disorders
Source: Brain. 2014 Jun 19;137(8):2382–95. doi: 10.1093/brain/awu132 (PMC4107735; doi:10.1093/brain/awu132)
Supplement: Supplementary Fig. 1 [file 8022a958cd852aa0c9fde0f243e1cdd7_brain-2013-01933-File010.docx]

**The hubs of the human connectome are generally implicated in the anatomy of brain disorders**

Nicolas A. Crossley, Andrea Mechelli, Jessica Scott, Francesco Carletti, Peter T. Fox, Philip McGuire and Edward T. Bullmore.

**Supplementary Information**

**Full list of studies included for each disorder**

**ATTENTION DEFICIT HYPERACTIVITY DISORDER**

([Overmeyer *et al.*, 2001](#_ENREF_230); [Carmona *et al.*, 2005](#_ENREF_63); [Brieber *et al.*, 2007](#_ENREF_51); [McAlonan *et al.*, 2007](#_ENREF_198); [Wang *et al.*, 2007](#_ENREF_320); [Yang *et al.*, 2008](#_ENREF_339); [Almeida Montes *et al.*, 2010](#_ENREF_11); [Depue *et al.*, 2010](#_ENREF_87); [Kobel *et al.*, 2010](#_ENREF_166); [Sasayama *et al.*, 2010](#_ENREF_268); [Ahrendts *et al.*, 2011](#_ENREF_8); [Amico *et al.*, 2011](#_ENREF_13); [Seidman *et al.*, 2011](#_ENREF_272))

**AMYOTROPHIC LATERAL SCLEROSIS**

([Ellis *et al.*, 2001](#_ENREF_97); [Chang *et al.*, 2005](#_ENREF_69); [Grosskreutz *et al.*, 2006](#_ENREF_119); [Agosta *et al.*, 2007](#_ENREF_6); [Mezzapesa *et al.*, 2007](#_ENREF_208); [Thivard *et al.*, 2007](#_ENREF_299); [Senda *et al.*, 2011](#_ENREF_274); [Cosottini *et al.*, 2012](#_ENREF_78))

**ALZHEIMER’S DISEASE**

([Baron *et al.*, 2001](#_ENREF_24); [Frisoni *et al.*, 2002](#_ENREF_103); [Matsuda *et al.*, 2002](#_ENREF_195); [Busatto *et al.*, 2003](#_ENREF_58); [Brenneis *et al.*, 2004](#_ENREF_50); [Grossman *et al.*, 2004](#_ENREF_120); [Ishii *et al.*, 2005](#_ENREF_145); [Remy *et al.*, 2005](#_ENREF_253); [Zahn *et al.*, 2005](#_ENREF_344); [Baxter *et al.*, 2006](#_ENREF_26); [Bozzali *et al.*, 2006](#_ENREF_45); [Hirao *et al.*, 2006](#_ENREF_134); [Xie *et al.*, 2006](#_ENREF_337); [Di Paola *et al.*, 2007](#_ENREF_89); [Farrow *et al.*, 2007](#_ENREF_99); [Hamalainen *et al.*, 2007](#_ENREF_130); [Rabinovici *et al.*, 2007](#_ENREF_247); [Samuraki *et al.*, 2007](#_ENREF_265); [Whitwell *et al.*, 2007](#_ENREF_325); [Berlingeri *et al.*, 2008](#_ENREF_29); [Feldmann *et al.*, 2008](#_ENREF_100); [Hall *et al.*, 2008](#_ENREF_128); [Mazere *et al.*, 2008](#_ENREF_197); [Shiino *et al.*, 2008](#_ENREF_277); [Brambati *et al.*, 2009](#_ENREF_46); [Honea *et al.*, 2009](#_ENREF_138); [Migliaccio *et al.*, 2009](#_ENREF_210); [Raji *et al.*, 2009](#_ENREF_248); [Rami *et al.*, 2009](#_ENREF_249); [Waragai *et al.*, 2009](#_ENREF_321); [Guo *et al.*, 2010](#_ENREF_121); [Agosta *et al.*, 2011](#_ENREF_7); [Kim *et al.*, 2011](#_ENREF_164); [Miettinen *et al.*, 2011](#_ENREF_209))

**ANOREXIA NERVOSA**

([Wagner *et al.*, 2006](#_ENREF_317); [Muhlau *et al.*, 2007](#_ENREF_219); [Castro-Fornieles *et al.*, 2009](#_ENREF_66); [Joos *et al.*, 2010](#_ENREF_149); [Suchan *et al.*, 2010](#_ENREF_287); [Boghi *et al.*, 2011](#_ENREF_36); [Brooks *et al.*, 2011](#_ENREF_52); [Gaudio *et al.*, 2011](#_ENREF_107); [Friederich *et al.*, 2012](#_ENREF_102); [Mainz *et al.*, 2012](#_ENREF_190))

**AUTISM**

([Boddaert *et al.*, 2004](#_ENREF_34); [Kwon *et al.*, 2004](#_ENREF_173); [McAlonan *et al.*, 2005](#_ENREF_199); [Rojas *et al.*, 2006](#_ENREF_257); [Bonilha *et al.*, 2008](#_ENREF_37); [Ke *et al.*, 2008](#_ENREF_156); [Wilson *et al.*, 2009](#_ENREF_330); [Hyde *et al.*, 2010](#_ENREF_142); [Toal *et al.*, 2010](#_ENREF_303); [Kurth *et al.*, 2011](#_ENREF_172); [Calderoni *et al.*, 2012](#_ENREF_59); [Ecker *et al.*, 2012](#_ENREF_94))

**ASPERGER SYNDROME**

([Abell *et al.*, 1999](#_ENREF_2); [McAlonan *et al.*, 2002](#_ENREF_200); [Kwon *et al.*, 2004](#_ENREF_173); [Salmond *et al.*, 2005](#_ENREF_263); [Brieber *et al.*, 2007](#_ENREF_51); [Craig *et al.*, 2007](#_ENREF_79); [McAlonan *et al.*, 2008](#_ENREF_201); [Ecker *et al.*, 2010](#_ENREF_93); [Toal *et al.*, 2010](#_ENREF_303))

**HEREDITARY ATAXIA**

([Brenneis *et al.*, 2003](#_ENREF_48); [Lasek *et al.*, 2006](#_ENREF_176); [Lukas *et al.*, 2006](#_ENREF_188); [Della Nave *et al.*, 2008](#_ENREF_84); [Della Nave *et al.*, 2008](#_ENREF_85); [Franca *et al.*, 2009](#_ENREF_101); [Reetz *et al.*, 2010](#_ENREF_252); [Alcauter *et al.*, 2011](#_ENREF_9); [D'Agata *et al.*, 2011](#_ENREF_81); [Goel *et al.*, 2011](#_ENREF_114); [Kirchner *et al.*, 2011](#_ENREF_165); [Reetz *et al.*, 2011](#_ENREF_251))

**BIPOLAR DISORDER**

([Doris *et al.*, 2004](#_ENREF_90); [Lochhead *et al.*, 2004](#_ENREF_186); [Lyoo *et al.*, 2004](#_ENREF_189); [McIntosh *et al.*, 2004](#_ENREF_202); [Adler *et al.*, 2005](#_ENREF_4); [Nugent *et al.*, 2006](#_ENREF_226); [Chen *et al.*, 2007](#_ENREF_71); [Yatham *et al.*, 2007](#_ENREF_341); [Haldane *et al.*, 2008](#_ENREF_127); [Almeida *et al.*, 2009](#_ENREF_10); [Ha *et al.*, 2009](#_ENREF_124); [Stanfield *et al.*, 2009](#_ENREF_285); [Cui *et al.*, 2011](#_ENREF_80); [Li *et al.*, 2011](#_ENREF_183); [Molina *et al.*, 2011](#_ENREF_211); [Wang *et al.*, 2011](#_ENREF_319); [Watson *et al.*, 2012](#_ENREF_322))

**DEMENTIA in PARKINSON'S DISEASE / LEWY BODY**

([Burton *et al.*, 2002](#_ENREF_56); [Burton *et al.*, 2004](#_ENREF_57); [Nagano-Saito *et al.*, 2005](#_ENREF_222); [Summerfield *et al.*, 2005](#_ENREF_288); [Beyer *et al.*, 2007](#_ENREF_32); [Sanchez-Castaneda *et al.*, 2009](#_ENREF_266); [Takahashi *et al.*, 2010](#_ENREF_295); [Ash *et al.*, 2011](#_ENREF_20); [Song *et al.*, 2011](#_ENREF_281))

**DEPRESSION**

([Shah *et al.*, 1998](#_ENREF_275); [Tang *et al.*, 2007](#_ENREF_296); [Frodl *et al.*, 2008](#_ENREF_104); [Kim *et al.*, 2008](#_ENREF_163); [Vasic *et al.*, 2008](#_ENREF_315); [Wagner *et al.*, 2008](#_ENREF_318); [Arnone *et al.*, 2009](#_ENREF_17); [Bergouignan *et al.*, 2009](#_ENREF_28); [Leung *et al.*, 2009](#_ENREF_180); [Treadway *et al.*, 2009](#_ENREF_306); [Zhang *et al.*, 2009](#_ENREF_347); [Abe *et al.*, 2010](#_ENREF_1); [Cheng *et al.*, 2010](#_ENREF_72); [Lai *et al.*, 2010](#_ENREF_174); [Li *et al.*, 2010](#_ENREF_181); [Scheuerecker *et al.*, 2010](#_ENREF_269); [van Tol *et al.*, 2010](#_ENREF_313); [Zou *et al.*, 2010](#_ENREF_348); [Amico *et al.*, 2011](#_ENREF_12); [Inkster *et al.*, 2011](#_ENREF_144); [Lee *et al.*, 2011](#_ENREF_178); [Peng *et al.*, 2011](#_ENREF_238); [Salvadore *et al.*, 2011](#_ENREF_264); [Soriano-Mas *et al.*, 2011](#_ENREF_283))

**DEVELOPMENTAL DYSLEXIA**

([Brown *et al.*, 2001](#_ENREF_53); [Brambati *et al.*, 2004](#_ENREF_47); [Eckert *et al.*, 2005](#_ENREF_95); [Silani *et al.*, 2005](#_ENREF_279); [Hoeft *et al.*, 2007](#_ENREF_135); [Kronbichler *et al.*, 2008](#_ENREF_170); [Menghini *et al.*, 2008](#_ENREF_205); [Steinbrink *et al.*, 2008](#_ENREF_286))

**DYSTONIA**

([Draganski *et al.*, 2003](#_ENREF_91); [Garraux *et al.*, 2004](#_ENREF_106); [Etgen *et al.*, 2006](#_ENREF_98); [Delmaire *et al.*, 2007](#_ENREF_86); [Egger *et al.*, 2007](#_ENREF_96); [Obermann *et al.*, 2007](#_ENREF_228); [Granert *et al.*, 2011](#_ENREF_118); [Martino *et al.*, 2011](#_ENREF_192); [Pantano *et al.*, 2011](#_ENREF_234); [Suzuki *et al.*, 2011](#_ENREF_290))

**FRONTOTEMPORAL DEMENTIA**

([Mummery *et al.*, 2000](#_ENREF_221); [Rosen *et al.*, 2002](#_ENREF_258); [Boxer *et al.*, 2003](#_ENREF_44); [Gee *et al.*, 2003](#_ENREF_108); [Sonty *et al.*, 2003](#_ENREF_282); [Gorno-Tempini *et al.*, 2004](#_ENREF_116); [Grossman *et al.*, 2004](#_ENREF_120); [Halpern *et al.*, 2004](#_ENREF_129); [Whitwell *et al.*, 2004](#_ENREF_328); [Boccardi *et al.*, 2005](#_ENREF_33); [Whitwell *et al.*, 2005](#_ENREF_326); [Zahn *et al.*, 2005](#_ENREF_344); [Adlam *et al.*, 2006](#_ENREF_3); [Gorno-Tempini *et al.*, 2006](#_ENREF_117); [Desgranges *et al.*, 2007](#_ENREF_88); [Kim *et al.*, 2007](#_ENREF_160); [Rabinovici *et al.*, 2007](#_ENREF_247); [Whitwell *et al.*, 2007](#_ENREF_327); [Kanda *et al.*, 2008](#_ENREF_151); [Seeley *et al.*, 2008](#_ENREF_271); [Zamboni *et al.*, 2008](#_ENREF_345); [Ash *et al.*, 2009](#_ENREF_21); [Libon *et al.*, 2009](#_ENREF_184); [Massimo *et al.*, 2009](#_ENREF_194); [Pardini *et al.*, 2009](#_ENREF_235); [Pereira *et al.*, 2009](#_ENREF_240); [Whitwell *et al.*, 2009](#_ENREF_324); [Wilson *et al.*, 2009](#_ENREF_331); [Wilson *et al.*, 2010](#_ENREF_332))

**HUNTINGTON’S DISEASE**

([Kassubek *et al.*, 2004](#_ENREF_154); [Peinemann *et al.*, 2005](#_ENREF_236); [Muhlau *et al.*, 2007](#_ENREF_220); [Beste *et al.*, 2008](#_ENREF_31); [Gomez-Anson *et al.*, 2009](#_ENREF_115); [Henley *et al.*, 2009](#_ENREF_131); [Wolf *et al.*, 2009](#_ENREF_335); [Ille *et al.*, 2011](#_ENREF_143); [Wolf *et al.*, 2011](#_ENREF_334))

**JUVENILE MYOCLONIC EPILEPSY**

([Tae *et al.*, 2006](#_ENREF_292); [Kim *et al.*, 2007](#_ENREF_161); [de Araujo Filho *et al.*, 2009](#_ENREF_82); [Lin *et al.*, 2009](#_ENREF_185); [Roebling *et al.*, 2009](#_ENREF_256); [Mory *et al.*, 2011](#_ENREF_218); [O'Muircheartaigh *et al.*, 2011](#_ENREF_227))

**MULTIPLE SCLEROSIS**

([Audoin *et al.*, 2006](#_ENREF_22); [Morgen *et al.*, 2006](#_ENREF_214); [Prinster *et al.*, 2006](#_ENREF_244); [Henry *et al.*, 2008](#_ENREF_132); [Mesaros *et al.*, 2008](#_ENREF_207); [Bodini *et al.*, 2009](#_ENREF_35); [Ceccarelli *et al.*, 2009](#_ENREF_67); [Audoin *et al.*, 2010](#_ENREF_23); [Prinster *et al.*, 2010](#_ENREF_243); [Spano *et al.*, 2010](#_ENREF_284); [Riccitelli *et al.*, 2012](#_ENREF_254))

**OBSESSIVE COMPULSIVE DISORDER**

([Kim *et al.*, 2001](#_ENREF_162); [Pujol *et al.*, 2004](#_ENREF_245); [Valente *et al.*, 2005](#_ENREF_310); [Carmona *et al.*, 2007](#_ENREF_62); [Gilbert *et al.*, 2008](#_ENREF_111); [Gilbert *et al.*, 2008](#_ENREF_112); [Szeszko *et al.*, 2008](#_ENREF_291); [Yoo *et al.*, 2008](#_ENREF_343); [Koprivova *et al.*, 2009](#_ENREF_167); [Lazaro *et al.*, 2009](#_ENREF_177); [van den Heuvel *et al.*, 2009](#_ENREF_312); [Matsumoto *et al.*, 2010](#_ENREF_196); [Togao *et al.*, 2010](#_ENREF_304); [Hoexter *et al.*, 2012](#_ENREF_136))

**OBSTRUCTIVE SLEEP APNEA**

([Morrell *et al.*, 2003](#_ENREF_217); [Celle *et al.*, 2009](#_ENREF_68); [Yaouhi *et al.*, 2009](#_ENREF_340); [Joo *et al.*, 2010](#_ENREF_148); [Morrell *et al.*, 2010](#_ENREF_216); [Canessa *et al.*, 2011](#_ENREF_61); [Torelli *et al.*, 2011](#_ENREF_305))

**CHRONIC PAIN**

([Apkarian *et al.*, 2004](#_ENREF_16); [Schmidt-Wilcke *et al.*, 2007](#_ENREF_270); [Buckalew *et al.*, 2008](#_ENREF_55); [Geha *et al.*, 2008](#_ENREF_109); [Hsu *et al.*, 2009](#_ENREF_140); [Valet *et al.*, 2009](#_ENREF_311); [Vartiainen *et al.*, 2009](#_ENREF_314); [Gwilym *et al.*, 2010](#_ENREF_123); [Seminowicz *et al.*, 2010](#_ENREF_273); [Tu *et al.*, 2010](#_ENREF_308); [Gerstner *et al.*, 2011](#_ENREF_110); [Gustin *et al.*, 2011](#_ENREF_122); [Ruscheweyh *et al.*, 2011](#_ENREF_259))

**PANIC DISORDER**

([Massana *et al.*, 2003](#_ENREF_193); [Yoo *et al.*, 2005](#_ENREF_342); [Uchida *et al.*, 2008](#_ENREF_309); [Asami *et al.*, 2009](#_ENREF_19); [Lai *et al.*, 2010](#_ENREF_174); [Sobanski *et al.*, 2010](#_ENREF_280); [Lai and Wu, 2012](#_ENREF_175))

**PARKINSON'S DISEASE**

([Burton *et al.*, 2004](#_ENREF_57); [Cordato *et al.*, 2005](#_ENREF_76); [Nagano-Saito *et al.*, 2005](#_ENREF_222); [Summerfield *et al.*, 2005](#_ENREF_288); [Ramirez-Ruiz *et al.*, 2007](#_ENREF_250); [Camicioli *et al.*, 2009](#_ENREF_60); [Jubault *et al.*, 2009](#_ENREF_150); [Pereira *et al.*, 2009](#_ENREF_239); [Tir *et al.*, 2009](#_ENREF_302); [Kostic *et al.*, 2010](#_ENREF_168); [Nishio *et al.*, 2010](#_ENREF_225); [Meppelink *et al.*, 2011](#_ENREF_206); [Morgen *et al.*, 2011](#_ENREF_215); [Melzer *et al.*, 2012](#_ENREF_204))

**PROGRESSIVE SUPRANUCLEAR PALSY**

([Brenneis *et al.*, 2004](#_ENREF_49); [Cordato *et al.*, 2005](#_ENREF_76); [Boxer *et al.*, 2006](#_ENREF_43); [Padovani *et al.*, 2006](#_ENREF_231); [Agosta *et al.*, 2010](#_ENREF_5); [Lehericy *et al.*, 2010](#_ENREF_179); [Takahashi *et al.*, 2011](#_ENREF_294))

**POST-TRAUMATIC STRESS DISORDER**

([Yamasue *et al.*, 2003](#_ENREF_338); [Corbo *et al.*, 2005](#_ENREF_75); [Chen *et al.*, 2006](#_ENREF_70); [Jatzko *et al.*, 2006](#_ENREF_146); [Li *et al.*, 2006](#_ENREF_182); [Hakamata *et al.*, 2007](#_ENREF_126); [Bryant *et al.*, 2008](#_ENREF_54); [Kasai *et al.*, 2008](#_ENREF_152); [Carrion *et al.*, 2009](#_ENREF_64); [Nardo *et al.*, 2010](#_ENREF_223); [Thomaes *et al.*, 2010](#_ENREF_300); [Eckart *et al.*, 2011](#_ENREF_92); [Zhang *et al.*, 2011](#_ENREF_346); [Tavanti *et al.*, 2012](#_ENREF_298))

**TEMPORAL LOBE EPILEPSY -left**

([Keller *et al.*, 2002](#_ENREF_159); [Bernasconi *et al.*, 2004](#_ENREF_30); [Bonilha *et al.*, 2004](#_ENREF_39); [McMillan *et al.*, 2004](#_ENREF_203); [Cormack *et al.*, 2005](#_ENREF_77); [Keller *et al.*, 2007](#_ENREF_158); [Bouilleret *et al.*, 2008](#_ENREF_42); [Pell *et al.*, 2008](#_ENREF_237); [Riederer *et al.*, 2008](#_ENREF_255); [Keller *et al.*, 2009](#_ENREF_157); [Pail *et al.*, 2010](#_ENREF_232); [Santana *et al.*, 2010](#_ENREF_267); [Tae *et al.*, 2010](#_ENREF_293))

**TEMPORAL LOBE EPILEPSY -right**

([Keller *et al.*, 2002](#_ENREF_159); [Bernasconi *et al.*, 2004](#_ENREF_30); [Bonilha *et al.*, 2004](#_ENREF_39); [McMillan *et al.*, 2004](#_ENREF_203); [Cormack *et al.*, 2005](#_ENREF_77); [Riederer *et al.*, 2008](#_ENREF_255); [Keller *et al.*, 2009](#_ENREF_157); [Pail *et al.*, 2010](#_ENREF_232); [Santana *et al.*, 2010](#_ENREF_267); [Tae *et al.*, 2010](#_ENREF_293))

**SCHIZOPHRENIA**

([Wright *et al.*, 1999](#_ENREF_336); [Hulshoff Pol *et al.*, 2001](#_ENREF_141); [Paillere-Martinot *et al.*, 2001](#_ENREF_233); [Sigmundsson *et al.*, 2001](#_ENREF_278); [Wilke *et al.*, 2001](#_ENREF_329); [Ananth *et al.*, 2002](#_ENREF_14); [Job *et al.*, 2002](#_ENREF_147); [Kubicki *et al.*, 2002](#_ENREF_171); [Shapleske *et al.*, 2002](#_ENREF_276); [Suzuki *et al.*, 2002](#_ENREF_289); [Marcelis *et al.*, 2003](#_ENREF_191); [Salgado-Pineda *et al.*, 2003](#_ENREF_260); [Ha *et al.*, 2004](#_ENREF_125); [McIntosh *et al.*, 2004](#_ENREF_202); [Moorhead *et al.*, 2004](#_ENREF_213); [Salgado-Pineda *et al.*, 2004](#_ENREF_262); [Antonova *et al.*, 2005](#_ENREF_15); [Giuliani *et al.*, 2005](#_ENREF_113); [Neckelmann *et al.*, 2006](#_ENREF_224); [Whitford *et al.*, 2006](#_ENREF_323); [Bassitt *et al.*, 2007](#_ENREF_25); [Chua *et al.*, 2007](#_ENREF_73); [Tregellas *et al.*, 2007](#_ENREF_307); [Bonilha *et al.*, 2008](#_ENREF_38); [Cooke *et al.*, 2008](#_ENREF_74); [Garcia-Marti *et al.*, 2008](#_ENREF_105); [Honea *et al.*, 2008](#_ENREF_137); [Koutsouleris *et al.*, 2008](#_ENREF_169); [Venkatasubramanian *et al.*, 2008](#_ENREF_316); [Bose *et al.*, 2009](#_ENREF_41); [Herold *et al.*, 2009](#_ENREF_133); [Kasparek *et al.*, 2009](#_ENREF_153); [Kawada *et al.*, 2009](#_ENREF_155); [Lui *et al.*, 2009](#_ENREF_187); [Witthaus *et al.*, 2009](#_ENREF_333); [Borgwardt *et al.*, 2010](#_ENREF_40); [Cascella *et al.*, 2010](#_ENREF_65); [Horn *et al.*, 2010](#_ENREF_139); [Molina *et al.*, 2010](#_ENREF_212); [Pomarol-Clotet *et al.*, 2010](#_ENREF_241); [Price *et al.*, 2010](#_ENREF_242); [Tanskanen *et al.*, 2010](#_ENREF_297); [Berge *et al.*, 2011](#_ENREF_27); [Cui *et al.*, 2011](#_ENREF_80); [de Castro-Manglano *et al.*, 2011](#_ENREF_83); [Ortiz-Gil *et al.*, 2011](#_ENREF_229); [Qiu *et al.*, 2011](#_ENREF_246); [Salgado-Pineda *et al.*, 2011](#_ENREF_261); [Tian *et al.*, 2011](#_ENREF_301); [Asami *et al.*, 2012](#_ENREF_18); [Watson *et al.*, 2012](#_ENREF_322))

**Full references**

Abe O, Yamasue H, Kasai K, Yamada H, Aoki S, Inoue H, et al. Voxel-based analyses of gray/white matter volume and diffusion tensor data in major depression. Psychiatry Research. 2010;181(1):64-70.

Abell F, Krams M, Ashburner J, Passingham R, Friston K, Frackowiak R, et al. The neuroanatomy of autism: a voxel-based whole brain analysis of structural scans. Neuroreport. 1999;10(8):1647-51.

Adlam AL, Patterson K, Rogers TT, Nestor PJ, Salmond CH, Acosta-Cabronero J, et al. Semantic dementia and fluent primary progressive aphasia: two sides of the same coin? Brain : a journal of neurology. 2006;129(Pt 11):3066-80.

Adler CM, Levine AD, DelBello MP, Strakowski SM. Changes in gray matter volume in patients with bipolar disorder. Biological Psychiatry. 2005;58(2):151-7.

Agosta F, Kostic VS, Galantucci S, Mesaros S, Svetel M, Pagani E, et al. The in vivo distribution of brain tissue loss in Richardson's syndrome and PSP-parkinsonism: a VBM-DARTEL study. Eur J Neurosci. 2010;32(4):640-7.

Agosta F, Pagani E, Rocca MA, Caputo D, Perini M, Salvi F, et al. Voxel-based morphometry study of brain volumetry and diffusivity in amyotrophic lateral sclerosis patients with mild disability. Human Brain Mapping. 2007;28(12):1430-8.

Agosta F, Pievani M, Sala S, Geroldi C, Galluzzi S, Frisoni GB, et al. White matter damage in Alzheimer disease and its relationship to gray matter atrophy. Radiology. 2011;258(3):853-63.

Ahrendts J, Rusch N, Wilke M, Philipsen A, Eickhoff SB, Glauche V, et al. Visual cortex abnormalities in adults with ADHD: a structural MRI study. World J Biol Psychiatry. 2011;12(4):260-70.

Alcauter S, Barrios FA, Diaz R, Fernandez-Ruiz J. Gray and white matter alterations in spinocerebellar ataxia type 7: an in vivo DTI and VBM study. NeuroImage. 2011;55(1):1-7.

Almeida JR, Akkal D, Hassel S, Travis MJ, Banihashemi L, Kerr N, et al. Reduced gray matter volume in ventral prefrontal cortex but not amygdala in bipolar disorder: significant effects of gender and trait anxiety. Psychiatry Research. 2009;171(1):54-68.

Almeida Montes LG, Ricardo-Garcell J, Barajas De La Torre LB, Prado Alcantara H, Martinez Garcia RB, Fernandez-Bouzas A, et al. Clinical correlations of grey matter reductions in the caudate nucleus of adults with attention deficit hyperactivity disorder. Journal of psychiatry & neuroscience : JPN. 2010;35(4):238-46.

Amico F, Meisenzahl E, Koutsouleris N, Reiser M, Moller HJ, Frodl T. Structural MRI correlates for vulnerability and resilience to major depressive disorder. Journal of psychiatry & neuroscience : JPN. 2011;36(1):15-22.

Amico F, Stauber J, Koutsouleris N, Frodl T. Anterior cingulate cortex gray matter abnormalities in adults with attention deficit hyperactivity disorder: a voxel-based morphometry study. Psychiatry Research. 2011;191(1):31-5.

Ananth H, Popescu I, Critchley HD, Good CD, Frackowiak RS, Dolan RJ. Cortical and subcortical gray matter abnormalities in schizophrenia determined through structural magnetic resonance imaging with optimized volumetric voxel-based morphometry. The American journal of psychiatry. 2002;159(9):1497-505.

Antonova E, Kumari V, Morris R, Halari R, Anilkumar A, Mehrotra R, et al. The relationship of structural alterations to cognitive deficits in schizophrenia: a voxel-based morphometry study. Biological Psychiatry. 2005;58(6):457-67.

Apkarian AV, Sosa Y, Sonty S, Levy RM, Harden RN, Parrish TB, et al. Chronic back pain is associated with decreased prefrontal and thalamic gray matter density. The Journal of neuroscience : the official journal of the Society for Neuroscience. 2004;24(46):10410-5.

Arnone D, Pegg EJ, Mckie S, Downey D, Elliot R, Williams SR, et al. P.2.a.019 Decreased fronto-limbic gray matter volume associated with recurrent major depressive disorder. European Neuropsychopharmacology. 2009;19:S371.

Asami T, Bouix S, Whitford TJ, Shenton ME, Salisbury DF, McCarley RW. Longitudinal loss of gray matter volume in patients with first-episode schizophrenia: DARTEL automated analysis and ROI validation. NeuroImage. 2012;59(2):986-96.

Asami T, Yamasue H, Hayano F, Nakamura M, Uehara K, Otsuka T, et al. Sexually dimorphic gray matter volume reduction in patients with panic disorder. Psychiatry Research. 2009;173(2):128-34.

Ash S, McMillan C, Gross RG, Cook P, Morgan B, Boller A, et al. The organization of narrative discourse in Lewy body spectrum disorder. Brain and language. 2011;119(1):30-41.

Ash S, Moore P, Vesely L, Gunawardena D, McMillan C, Anderson C, et al. Non-Fluent Speech in Frontotemporal Lobar Degeneration. J Neurolinguistics. 2009;22(4):370-83.

Audoin B, Davies GR, Finisku L, Chard DT, Thompson AJ, Miller DH. Localization of grey matter atrophy in early RRMS : A longitudinal study. J Neurol. 2006;253(11):1495-501.

Audoin B, Zaaraoui W, Reuter F, Rico A, Malikova I, Confort-Gouny S, et al. Atrophy mainly affects the limbic system and the deep grey matter at the first stage of multiple sclerosis. J Neurol Neurosurg Psychiatry. 2010;81(6):690-5.

Baron JC, Chetelat G, Desgranges B, Perchey G, Landeau B, de la Sayette V, et al. In vivo mapping of gray matter loss with voxel-based morphometry in mild Alzheimer's disease. NeuroImage. 2001;14(2):298-309.

Bassitt DP, Neto MR, de Castro CC, Busatto GF. Insight and regional brain volumes in schizophrenia. European Archives of Psychiatry and Clinical Neuroscience. 2007;257(1):58-62.

Baxter LC, Sparks DL, Johnson SC, Lenoski B, Lopez JE, Connor DJ, et al. Relationship of cognitive measures and gray and white matter in Alzheimer's disease. J Alzheimers Dis. 2006;9(3):253-60.

Berge D, Carmona S, Rovira M, Bulbena A, Salgado P, Vilarroya O. Gray matter volume deficits and correlation with insight and negative symptoms in first-psychotic-episode subjects. Acta Psychiatrica Scandinavica. 2011;123(6):431-9.

Bergouignan L, Chupin M, Czechowska Y, Kinkingnehun S, Lemogne C, Le Bastard G, et al. Can voxel based morphometry, manual segmentation and automated segmentation equally detect hippocampal volume differences in acute depression? NeuroImage. 2009;45(1):29-37.

Berlingeri M, Bottini G, Basilico S, Silani G, Zanardi G, Sberna M, et al. Anatomy of the episodic buffer: a voxel-based morphometry study in patients with dementia. Behav Neurol. 2008;19(1-2):29-34.

Bernasconi N, Duchesne S, Janke A, Lerch J, Collins DL, Bernasconi A. Whole-brain voxel-based statistical analysis of gray matter and white matter in temporal lobe epilepsy. NeuroImage. 2004;23(2):717-23.

Beste C, Saft C, Konrad C, Andrich J, Habbel A, Schepers I, et al. Levels of error processing in Huntington's disease: a combined study using event-related potentials and voxel-based morphometry. Human Brain Mapping. 2008;29(2):121-30.

Beyer MK, Janvin CC, Larsen JP, Aarsland D. A magnetic resonance imaging study of patients with Parkinson's disease with mild cognitive impairment and dementia using voxel-based morphometry. J Neurol Neurosurg Psychiatry. 2007;78(3):254-9.

Boccardi M, Sabattoli F, Laakso MP, Testa C, Rossi R, Beltramello A, et al. Frontotemporal dementia as a neural system disease. Neurobiology of Aging. 2005;26(1):37-44.

Boddaert N, De Leersnyder H, Bourgeois M, Munnich A, Brunelle F, Zilbovicius M. Anatomical and functional brain imaging evidence of lenticulo-insular anomalies in Smith Magenis syndrome. NeuroImage. 2004;21(3):1021-5.

Bodini B, Khaleeli Z, Cercignani M, Miller DH, Thompson AJ, Ciccarelli O. Exploring the relationship between white matter and gray matter damage in early primary progressive multiple sclerosis: an in vivo study with TBSS and VBM. Human Brain Mapping. 2009;30(9):2852-61.

Boghi A, Sterpone S, Sales S, D'Agata F, Bradac GB, Zullo G, et al. In vivo evidence of global and focal brain alterations in anorexia nervosa. Psychiatry Research. 2011;192(3):154-9.

Bonilha L, Cendes F, Rorden C, Eckert M, Dalgalarrondo P, Li LM, et al. Gray and white matter imbalance--typical structural abnormality underlying classic autism? Brain Dev. 2008;30(6):396-401.

Bonilha L, Molnar C, Horner MD, Anderson B, Forster L, George MS, et al. Neurocognitive deficits and prefrontal cortical atrophy in patients with schizophrenia. Schizophrenia Research. 2008;101(1-3):142-51.

Bonilha L, Rorden C, Castellano G, Pereira F, Rio PA, Cendes F, et al. Voxel-based morphometry reveals gray matter network atrophy in refractory medial temporal lobe epilepsy. Arch Neurol. 2004;61(9):1379-84.

Borgwardt SJ, Picchioni MM, Ettinger U, Toulopoulou T, Murray R, McGuire PK. Regional gray matter volume in monozygotic twins concordant and discordant for schizophrenia. Biological Psychiatry. 2010;67(10):956-64.

Bose SK, Mackinnon T, Mehta MA, Turkheimer FE, Howes OD, Selvaraj S, et al. The effect of ageing on grey and white matter reductions in schizophrenia. Schizophrenia Research. 2009;112(1-3):7-13.

Bouilleret V, Semah F, Chassoux F, Mantzaridez M, Biraben A, Trebossen R, et al. Basal ganglia involvement in temporal lobe epilepsy: a functional and morphologic study. Neurology. 2008;70(3):177-84.

Boxer AL, Geschwind MD, Belfor N, Gorno-Tempini ML, Schauer GF, Miller BL, et al. Patterns of brain atrophy that differentiate corticobasal degeneration syndrome from progressive supranuclear palsy. Arch Neurol. 2006;63(1):81-6.

Boxer AL, Rankin KP, Miller BL, Schuff N, Weiner M, Gorno-Tempini ML, et al. Cinguloparietal atrophy distinguishes Alzheimer disease from semantic dementia. Arch Neurol. 2003;60(7):949-56.

Bozzali M, Filippi M, Magnani G, Cercignani M, Franceschi M, Schiatti E, et al. The contribution of voxel-based morphometry in staging patients with mild cognitive impairment. Neurology. 2006;67(3):453-60.

Brambati SM, Belleville S, Kergoat MJ, Chayer C, Gauthier S, Joubert S. Single- and multiple-domain amnestic mild cognitive impairment: two sides of the same coin? Dement Geriatr Cogn Disord. 2009;28(6):541-9.

Brambati SM, Termine C, Ruffino M, Stella G, Fazio F, Cappa SF, et al. Regional reductions of gray matter volume in familial dyslexia. Neurology. 2004;63(4):742-5.

Brenneis C, Bosch SM, Schocke M, Wenning GK, Poewe W. Atrophy pattern in SCA2 determined by voxel-based morphometry. Neuroreport. 2003;14(14):1799-802.

Brenneis C, Seppi K, Schocke M, Benke T, Wenning GK, Poewe W. Voxel based morphometry reveals a distinct pattern of frontal atrophy in progressive supranuclear palsy. J Neurol Neurosurg Psychiatry. 2004;75(2):246-9.

Brenneis C, Wenning GK, Egger KE, Schocke M, Trieb T, Seppi K, et al. Basal forebrain atrophy is a distinctive pattern in dementia with Lewy bodies. Neuroreport. 2004;15(11):1711-4.

Brieber S, Neufang S, Bruning N, Kamp-Becker I, Remschmidt H, Herpertz-Dahlmann B, et al. Structural brain abnormalities in adolescents with autism spectrum disorder and patients with attention deficit/hyperactivity disorder. J Child Psychol Psychiatry. 2007;48(12):1251-8.

Brooks SJ, Barker GJ, O'Daly OG, Brammer M, Williams SC, Benedict C, et al. Restraint of appetite and reduced regional brain volumes in anorexia nervosa: a voxel-based morphometric study. BMC Psychiatry. 2011;11:179.

Brown WE, Eliez S, Menon V, Rumsey JM, White CD, Reiss AL. Preliminary evidence of widespread morphological variations of the brain in dyslexia. Neurology. 2001;56(6):781-3.

Bryant RA, Felmingham K, Whitford TJ, Kemp A, Hughes G, Peduto A, et al. Rostral anterior cingulate volume predicts treatment response to cognitive-behavioural therapy for posttraumatic stress disorder. Journal of psychiatry & neuroscience : JPN. 2008;33(2):142-6.

Buckalew N, Haut MW, Morrow L, Weiner D. Chronic pain is associated with brain volume loss in older adults: preliminary evidence. Pain Med. 2008;9(2):240-8.

Burton EJ, Karas G, Paling SM, Barber R, Williams ED, Ballard CG, et al. Patterns of cerebral atrophy in dementia with Lewy bodies using voxel-based morphometry. NeuroImage. 2002;17(2):618-30.

Burton EJ, McKeith IG, Burn DJ, Williams ED, O'Brien JT. Cerebral atrophy in Parkinson's disease with and without dementia: a comparison with Alzheimer's disease, dementia with Lewy bodies and controls. Brain : a journal of neurology. 2004;127(Pt 4):791-800.

Busatto GF, Garrido GE, Almeida OP, Castro CC, Camargo CH, Cid CG, et al. A voxel-based morphometry study of temporal lobe gray matter reductions in Alzheimer's disease. Neurobiology of Aging. 2003;24(2):221-31.

Calderoni S, Retico A, Biagi L, Tancredi R, Muratori F, Tosetti M. Female children with autism spectrum disorder: an insight from mass-univariate and pattern classification analyses. NeuroImage. 2012;59(2):1013-22.

Camicioli R, Gee M, Bouchard TP, Fisher NJ, Hanstock CC, Emery DJ, et al. Voxel-based morphometry reveals extra-nigral atrophy patterns associated with dopamine refractory cognitive and motor impairment in parkinsonism. Parkinsonism Relat Disord. 2009;15(3):187-95.

Canessa N, Castronovo V, Cappa SF, Aloia MS, Marelli S, Falini A, et al. Obstructive sleep apnea: brain structural changes and neurocognitive function before and after treatment. Am J Respir Crit Care Med. 2011;183(10):1419-26.

Carmona S, Bassas N, Rovira M, Gispert JD, Soliva JC, Prado M, et al. Pediatric OCD structural brain deficits in conflict monitoring circuits: a voxel-based morphometry study. Neuroscience letters. 2007;421(3):218-23.

Carmona S, Vilarroya O, Bielsa A, Tremols V, Soliva JC, Rovira M, et al. Global and regional gray matter reductions in ADHD: a voxel-based morphometric study. Neuroscience letters. 2005;389(2):88-93.

Carrion VG, Weems CF, Watson C, Eliez S, Menon V, Reiss AL. Converging evidence for abnormalities of the prefrontal cortex and evaluation of midsagittal structures in pediatric posttraumatic stress disorder: an MRI study. Psychiatry Research. 2009;172(3):226-34.

Cascella NG, Fieldstone SC, Rao VA, Pearlson GD, Sawa A, Schretlen DJ. Gray-matter abnormalities in deficit schizophrenia. Schizophrenia Research. 2010;120(1-3):63-70.

Castro-Fornieles J, Bargallo N, Lazaro L, Andres S, Falcon C, Plana MT, et al. A cross-sectional and follow-up voxel-based morphometric MRI study in adolescent anorexia nervosa. Journal of Psychiatric Research. 2009;43(3):331-40.

Ceccarelli A, Rocca MA, Valsasina P, Rodegher M, Pagani E, Falini A, et al. A multiparametric evaluation of regional brain damage in patients with primary progressive multiple sclerosis. Human Brain Mapping. 2009;30(9):3009-19.

Celle S, Peyron R, Faillenot I, Pichot V, Alabdullah M, Gaspoz JM, et al. Undiagnosed sleep-related breathing disorders are associated with focal brainstem atrophy in the elderly. Human Brain Mapping. 2009;30(7):2090-7.

Chang JL, Lomen-Hoerth C, Murphy J, Henry RG, Kramer JH, Miller BL, et al. A voxel-based morphometry study of patterns of brain atrophy in ALS and ALS/FTLD. Neurology. 2005;65(1):75-80.

Chen S, Xia W, Li L, Liu J, He Z, Zhang Z, et al. Gray matter density reduction in the insula in fire survivors with posttraumatic stress disorder: a voxel-based morphometric study. Psychiatry Research. 2006;146(1):65-72.

Chen X, Wen W, Malhi GS, Ivanovski B, Sachdev PS. Regional gray matter changes in bipolar disorder: a voxel-based morphometric study. The Australian and New Zealand journal of psychiatry. 2007;41(4):327-36.

Cheng YQ, Xu J, Chai P, Li HJ, Luo CR, Yang T, et al. Brain volume alteration and the correlations with the clinical characteristics in drug-naive first-episode MDD patients: a voxel-based morphometry study. Neuroscience letters. 2010;480(1):30-4.

Chua SE, Cheung C, Cheung V, Tsang JT, Chen EY, Wong JC, et al. Cerebral grey, white matter and csf in never-medicated, first-episode schizophrenia. Schizophrenia Research. 2007;89(1-3):12-21.

Cooke MA, Fannon D, Kuipers E, Peters E, Williams SC, Kumari V. Neurological basis of poor insight in psychosis: a voxel-based MRI study. Schizophrenia Research. 2008;103(1-3):40-51.

Corbo V, Clement MH, Armony JL, Pruessner JC, Brunet A. Size versus shape differences: contrasting voxel-based and volumetric analyses of the anterior cingulate cortex in individuals with acute posttraumatic stress disorder. Biological Psychiatry. 2005;58(2):119-24.

Cordato NJ, Duggins AJ, Halliday GM, Morris JG, Pantelis C. Clinical deficits correlate with regional cerebral atrophy in progressive supranuclear palsy. Brain : a journal of neurology. 2005;128(Pt 6):1259-66.

Cormack F, Gadian DG, Vargha-Khadem F, Cross JH, Connelly A, Baldeweg T. Extra-hippocampal grey matter density abnormalities in paediatric mesial temporal sclerosis. NeuroImage. 2005;27(3):635-43.

Cosottini M, Pesaresi I, Piazza S, Diciotti S, Cecchi P, Fabbri S, et al. Structural and functional evaluation of cortical motor areas in Amyotrophic Lateral Sclerosis. Exp Neurol. 2012;234(1):169-80.

Craig MC, Zaman SH, Daly EM, Cutter WJ, Robertson DM, Hallahan B, et al. Women with autistic-spectrum disorder: magnetic resonance imaging study of brain anatomy. The British journal of psychiatry : the journal of mental science. 2007;191:224-8.

Cui L, Li M, Deng W, Guo W, Ma X, Huang C, et al. Overlapping clusters of gray matter deficits in paranoid schizophrenia and psychotic bipolar mania with family history. Neuroscience letters. 2011;489(2):94-8.

D'Agata F, Caroppo P, Boghi A, Coriasco M, Caglio M, Baudino B, et al. Linking coordinative and executive dysfunctions to atrophy in spinocerebellar ataxia 2 patients. Brain Struct Funct. 2011;216(3):275-88.

de Araujo Filho GM, Jackowski AP, Lin K, Guaranha MS, Guilhoto LM, da Silva HH, et al. Personality traits related to juvenile myoclonic epilepsy: MRI reveals prefrontal abnormalities through a voxel-based morphometry study. Epilepsy Behav. 2009;15(2):202-7.

de Castro-Manglano P, Mechelli A, Soutullo C, Landecho I, Gimenez-Amaya JM, Ortuno F, et al. Structural brain abnormalities in first-episode psychosis: differences between affective psychoses and schizophrenia and relationship to clinical outcome. Bipolar disorders. 2011;13(5-6):545-55.

Della Nave R, Ginestroni A, Giannelli M, Tessa C, Salvatore E, Salvi F, et al. Brain structural damage in Friedreich's ataxia. J Neurol Neurosurg Psychiatry. 2008;79(1):82-5.

Della Nave R, Ginestroni A, Tessa C, Cosottini M, Giannelli M, Salvatore E, et al. Brain structural damage in spinocerebellar ataxia type 2. A voxel-based morphometry study. Mov Disord. 2008;23(6):899-903.

Delmaire C, Vidailhet M, Elbaz A, Bourdain F, Bleton JP, Sangla S, et al. Structural abnormalities in the cerebellum and sensorimotor circuit in writer's cramp. Neurology. 2007;69(4):376-80.

Depue BE, Burgess GC, Bidwell LC, Willcutt EG, Banich MT. Behavioral performance predicts grey matter reductions in the right inferior frontal gyrus in young adults with combined type ADHD. Psychiatry Research. 2010;182(3):231-7.

Desgranges B, Matuszewski V, Piolino P, Chetelat G, Mezenge F, Landeau B, et al. Anatomical and functional alterations in semantic dementia: a voxel-based MRI and PET study. Neurobiology of Aging. 2007;28(12):1904-13.

Di Paola M, Macaluso E, Carlesimo GA, Tomaiuolo F, Worsley KJ, Fadda L, et al. Episodic memory impairment in patients with Alzheimer's disease is correlated with entorhinal cortex atrophy. A voxel-based morphometry study. J Neurol. 2007;254(6):774-81.

Doris A, Belton E, Ebmeier KP, Glabus MF, Marshall I. Reduction of cingulate gray matter density in poor outcome bipolar illness. Psychiatry Research. 2004;130(2):153-9.

Draganski B, Thun-Hohenstein C, Bogdahn U, Winkler J, May A. "Motor circuit" gray matter changes in idiopathic cervical dystonia. Neurology. 2003;61(9):1228-31.

Eckart C, Stoppel C, Kaufmann J, Tempelmann C, Hinrichs H, Elbert T, et al. Structural alterations in lateral prefrontal, parietal and posterior midline regions of men with chronic posttraumatic stress disorder. Journal of psychiatry & neuroscience : JPN. 2011;36(3):176-86.

Ecker C, Rocha-Rego V, Johnston P, Mourao-Miranda J, Marquand A, Daly EM, et al. Investigating the predictive value of whole-brain structural MR scans in autism: a pattern classification approach. NeuroImage. 2010;49(1):44-56.

Ecker C, Suckling J, Deoni SC, Lombardo MV, Bullmore ET, Baron-Cohen S, et al. Brain anatomy and its relationship to behavior in adults with autism spectrum disorder: a multicenter magnetic resonance imaging study. Archives of general psychiatry. 2012;69(2):195-209.

Eckert MA, Leonard CM, Wilke M, Eckert M, Richards T, Richards A, et al. Anatomical signatures of dyslexia in children: unique information from manual and voxel based morphometry brain measures. Cortex; a journal devoted to the study of the nervous system and behavior. 2005;41(3):304-15.

Egger K, Mueller J, Schocke M, Brenneis C, Rinnerthaler M, Seppi K, et al. Voxel based morphometry reveals specific gray matter changes in primary dystonia. Mov Disord. 2007;22(11):1538-42.

Ellis CM, Suckling J, Amaro E, Jr., Bullmore ET, Simmons A, Williams SC, et al. Volumetric analysis reveals corticospinal tract degeneration and extramotor involvement in ALS. Neurology. 2001;57(9):1571-8.

Etgen T, Muhlau M, Gaser C, Sander D. Bilateral grey-matter increase in the putamen in primary blepharospasm. J Neurol Neurosurg Psychiatry. 2006;77(9):1017-20.

Farrow TF, Thiyagesh SN, Wilkinson ID, Parks RW, Ingram L, Woodruff PW. Fronto-temporal-lobe atrophy in early-stage Alzheimer's disease identified using an improved detection methodology. Psychiatry Research. 2007;155(1):11-9.

Feldmann A, Trauninger A, Toth L, Kotek G, Kosztolanyi P, Illes E, et al. Atrophy and decreased activation of fronto-parietal attention areas contribute to higher visual dysfunction in posterior cortical atrophy. Psychiatry Research. 2008;164(2):178-84.

Franca MC, Jr., D'Abreu A, Yasuda CL, Bonadia LC, Santos da Silva M, Nucci A, et al. A combined voxel-based morphometry and 1H-MRS study in patients with Friedreich's ataxia. J Neurol. 2009;256(7):1114-20.

Friederich HC, Walther S, Bendszus M, Biller A, Thomann P, Zeigermann S, et al. Grey matter abnormalities within cortico-limbic-striatal circuits in acute and weight-restored anorexia nervosa patients. NeuroImage. 2012;59(2):1106-13.

Frisoni GB, Testa C, Zorzan A, Sabattoli F, Beltramello A, Soininen H, et al. Detection of grey matter loss in mild Alzheimer's disease with voxel based morphometry. J Neurol Neurosurg Psychiatry. 2002;73(6):657-64.

Frodl T, Koutsouleris N, Bottlender R, Born C, Jager M, Morgenthaler M, et al. Reduced gray matter brain volumes are associated with variants of the serotonin transporter gene in major depression. Molecular Psychiatry. 2008;13(12):1093-101.

Garcia-Marti G, Aguilar EJ, Lull JJ, Marti-Bonmati L, Escarti MJ, Manjon JV, et al. Schizophrenia with auditory hallucinations: a voxel-based morphometry study. Prog Neuropsychopharmacol Biol Psychiatry. 2008;32(1):72-80.

Garraux G, Bauer A, Hanakawa T, Wu T, Kansaku K, Hallett M. Changes in brain anatomy in focal hand dystonia. Annals of neurology. 2004;55(5):736-9.

Gaudio S, Nocchi F, Franchin T, Genovese E, Cannata V, Longo D, et al. Gray matter decrease distribution in the early stages of Anorexia Nervosa restrictive type in adolescents. Psychiatry Research. 2011;191(1):24-30.

Gee J, Ding L, Xie Z, Lin M, DeVita C, Grossman M. Alzheimer's disease and frontotemporal dementia exhibit distinct atrophy-behavior correlates: a computer-assisted imaging study. Acad Radiol. 2003;10(12):1392-401.

Geha PY, Baliki MN, Harden RN, Bauer WR, Parrish TB, Apkarian AV. The brain in chronic CRPS pain: abnormal gray-white matter interactions in emotional and autonomic regions. Neuron. 2008;60(4):570-81.

Gerstner G, Ichesco E, Quintero A, Schmidt-Wilcke T. Changes in regional gray and white matter volume in patients with myofascial-type temporomandibular disorders: a voxel-based morphometry study. J Orofac Pain. 2011;25(2):99-106.

Gilbert AR, Keshavan MS, Diwadkar V, Nutche J, Macmaster F, Easter PC, et al. Gray matter differences between pediatric obsessive-compulsive disorder patients and high-risk siblings: a preliminary voxel-based morphometry study. Neuroscience letters. 2008;435(1):45-50.

Gilbert AR, Mataix-Cols D, Almeida JR, Lawrence N, Nutche J, Diwadkar V, et al. Brain structure and symptom dimension relationships in obsessive-compulsive disorder: a voxel-based morphometry study. J Affect Disord. 2008;109(1-2):117-26.

Giuliani NR, Calhoun VD, Pearlson GD, Francis A, Buchanan RW. Voxel-based morphometry versus region of interest: a comparison of two methods for analyzing gray matter differences in schizophrenia. Schizophrenia Research. 2005;74(2-3):135-47.

Goel G, Pal PK, Ravishankar S, Venkatasubramanian G, Jayakumar PN, Krishna N, et al. Gray matter volume deficits in spinocerebellar ataxia: an optimized voxel based morphometric study. Parkinsonism Relat Disord. 2011;17(7):521-7.

Gomez-Anson B, Alegret M, Munoz E, Monte GC, Alayrach E, Sanchez A, et al. Prefrontal cortex volume reduction on MRI in preclinical Huntington's disease relates to visuomotor performance and CAG number. Parkinsonism Relat Disord. 2009;15(3):213-9.

Gorno-Tempini ML, Dronkers NF, Rankin KP, Ogar JM, Phengrasamy L, Rosen HJ, et al. Cognition and anatomy in three variants of primary progressive aphasia. Annals of neurology. 2004;55(3):335-46.

Gorno-Tempini ML, Ogar JM, Brambati SM, Wang P, Jeong JH, Rankin KP, et al. Anatomical correlates of early mutism in progressive nonfluent aphasia. Neurology. 2006;67(10):1849-51.

Granert O, Peller M, Jabusch HC, Altenmuller E, Siebner HR. Sensorimotor skills and focal dystonia are linked to putaminal grey-matter volume in pianists. J Neurol Neurosurg Psychiatry. 2011;82(11):1225-31.

Grosskreutz J, Kaufmann J, Fradrich J, Dengler R, Heinze HJ, Peschel T. Widespread sensorimotor and frontal cortical atrophy in Amyotrophic Lateral Sclerosis. BMC Neurol. 2006;6:17.

Grossman M, McMillan C, Moore P, Ding L, Glosser G, Work M, et al. What's in a name: voxel-based morphometric analyses of MRI and naming difficulty in Alzheimer's disease, frontotemporal dementia and corticobasal degeneration. Brain : a journal of neurology. 2004;127(Pt 3):628-49.

Guo X, Wang Z, Li K, Li Z, Qi Z, Jin Z, et al. Voxel-based assessment of gray and white matter volumes in Alzheimer's disease. Neuroscience letters. 2010;468(2):146-50.

Gustin SM, Peck CC, Wilcox SL, Nash PG, Murray GM, Henderson LA. Different pain, different brain: thalamic anatomy in neuropathic and non-neuropathic chronic pain syndromes. The Journal of neuroscience : the official journal of the Society for Neuroscience. 2011;31(16):5956-64.

Gwilym SE, Filippini N, Douaud G, Carr AJ, Tracey I. Thalamic atrophy associated with painful osteoarthritis of the hip is reversible after arthroplasty: a longitudinal voxel-based morphometric study. Arthritis Rheum. 2010;62(10):2930-40.

Ha TH, Ha K, Kim JH, Choi JE. Regional brain gray matter abnormalities in patients with bipolar II disorder: a comparison study with bipolar I patients and healthy controls. Neuroscience letters. 2009;456(1):44-8.

Ha TH, Youn T, Ha KS, Rho KS, Lee JM, Kim IY, et al. Gray matter abnormalities in paranoid schizophrenia and their clinical correlations. Psychiatry Research. 2004;132(3):251-60.

Hakamata Y, Matsuoka Y, Inagaki M, Nagamine M, Hara E, Imoto S, et al. Structure of orbitofrontal cortex and its longitudinal course in cancer-related post-traumatic stress disorder. Neurosci Res. 2007;59(4):383-9.

Haldane M, Cunningham G, Androutsos C, Frangou S. Structural brain correlates of response inhibition in Bipolar Disorder I. Journal of Psychopharmacology. 2008;22(2):138-43.

Hall AM, Moore RY, Lopez OL, Kuller L, Becker JT. Basal forebrain atrophy is a presymptomatic marker for Alzheimer's disease. Alzheimers Dement. 2008;4(4):271-9.

Halpern CH, Glosser G, Clark R, Gee J, Moore P, Dennis K, et al. Dissociation of numbers and objects in corticobasal degeneration and semantic dementia. Neurology. 2004;62(7):1163-9.

Hamalainen A, Pihlajamaki M, Tanila H, Hanninen T, Niskanen E, Tervo S, et al. Increased fMRI responses during encoding in mild cognitive impairment. Neurobiology of Aging. 2007;28(12):1889-903.

Henley SM, Wild EJ, Hobbs NZ, Scahill RI, Ridgway GR, Macmanus DG, et al. Relationship between CAG repeat length and brain volume in premanifest and early Huntington's disease. J Neurol. 2009;256(2):203-12.

Henry RG, Shieh M, Okuda DT, Evangelista A, Gorno-Tempini ML, Pelletier D. Regional grey matter atrophy in clinically isolated syndromes at presentation. J Neurol Neurosurg Psychiatry. 2008;79(11):1236-44.

Herold R, Feldmann A, Simon M, Tenyi T, Kover F, Nagy F, et al. Regional gray matter reduction and theory of mind deficit in the early phase of schizophrenia: a voxel-based morphometric study. Acta Psychiatrica Scandinavica. 2009;119(3):199-208.

Hirao K, Ohnishi T, Matsuda H, Nemoto K, Hirata Y, Yamashita F, et al. Functional interactions between entorhinal cortex and posterior cingulate cortex at the very early stage of Alzheimer's disease using brain perfusion single-photon emission computed tomography. Nucl Med Commun. 2006;27(2):151-6.

Hoeft F, Meyler A, Hernandez A, Juel C, Taylor-Hill H, Martindale JL, et al. Functional and morphometric brain dissociation between dyslexia and reading ability. P Natl Acad Sci USA. 2007;104(10):4234-9.

Hoexter MQ, de Souza Duran FL, D'Alcante CC, Dougherty DD, Shavitt RG, Lopes AC, et al. Gray matter volumes in obsessive-compulsive disorder before and after fluoxetine or cognitive-behavior therapy: a randomized clinical trial. Neuropsychopharmacology : official publication of the American College of Neuropsychopharmacology. 2012;37(3):734-45.

Honea RA, Meyer-Lindenberg A, Hobbs KB, Pezawas L, Mattay VS, Egan MF, et al. Is gray matter volume an intermediate phenotype for schizophrenia? A voxel-based morphometry study of patients with schizophrenia and their healthy siblings. Biological Psychiatry. 2008;63(5):465-74.

Honea RA, Thomas GP, Harsha A, Anderson HS, Donnelly JE, Brooks WM, et al. Cardiorespiratory fitness and preserved medial temporal lobe volume in Alzheimer disease. Alzheimer Dis Assoc Disord. 2009;23(3):188-97.

Horn H, Federspiel A, Wirth M, Muller TJ, Wiest R, Walther S, et al. Gray matter volume differences specific to formal thought disorder in schizophrenia. Psychiatry Research. 2010;182(2):183-6.

Hsu MC, Harris RE, Sundgren PC, Welsh RC, Fernandes CR, Clauw DJ, et al. No consistent difference in gray matter volume between individuals with fibromyalgia and age-matched healthy subjects when controlling for affective disorder. Pain. 2009;143(3):262-7.

Hulshoff Pol HE, Schnack HG, Mandl RC, van Haren NE, Koning H, Collins DL, et al. Focal gray matter density changes in schizophrenia. Archives of general psychiatry. 2001;58(12):1118-25.

Hyde KL, Samson F, Evans AC, Mottron L. Neuroanatomical differences in brain areas implicated in perceptual and other core features of autism revealed by cortical thickness analysis and voxel-based morphometry. Human Brain Mapping. 2010;31(4):556-66.

Ille R, Schafer A, Scharmuller W, Enzinger C, Schoggl H, Kapfhammer HP, et al. Emotion recognition and experience in Huntington disease: a voxel-based morphometry study. Journal of psychiatry & neuroscience : JPN. 2011;36(6):383-90.

Inkster B, Rao AW, Ridler K, Nichols TE, Saemann PG, Auer DP, et al. Structural brain changes in patients with recurrent major depressive disorder presenting with anxiety symptoms. J Neuroimaging. 2011;21(4):375-82.

Ishii K, Kawachi T, Sasaki H, Kono AK, Fukuda T, Kojima Y, et al. Voxel-based morphometric comparison between early- and late-onset mild Alzheimer's disease and assessment of diagnostic performance of z score images. AJNR Am J Neuroradiol. 2005;26(2):333-40.

Jatzko A, Rothenhofer S, Schmitt A, Gaser C, Demirakca T, Weber-Fahr W, et al. Hippocampal volume in chronic posttraumatic stress disorder (PTSD): MRI study using two different evaluation methods. J Affect Disord. 2006;94(1-3):121-6.

Job DE, Whalley HC, McConnell S, Glabus M, Johnstone EC, Lawrie SM. Structural gray matter differences between first-episode schizophrenics and normal controls using voxel-based morphometry. NeuroImage. 2002;17(2):880-9.

Joo EY, Tae WS, Lee MJ, Kang JW, Park HS, Lee JY, et al. Reduced brain gray matter concentration in patients with obstructive sleep apnea syndrome. Sleep. 2010;33(2):235-41.

Joos A, Kloppel S, Hartmann A, Glauche V, Tuscher O, Perlov E, et al. Voxel-based morphometry in eating disorders: correlation of psychopathology with grey matter volume. Psychiatry Research. 2010;182(2):146-51.

Jubault T, Brambati SM, Degroot C, Kullmann B, Strafella AP, Lafontaine AL, et al. Regional brain stem atrophy in idiopathic Parkinson's disease detected by anatomical MRI. PLoS One. 2009;4(12):e8247.

Kanda T, Ishii K, Uemura T, Miyamoto N, Yoshikawa T, Kono AK, et al. Comparison of grey matter and metabolic reductions in frontotemporal dementia using FDG-PET and voxel-based morphometric MR studies. Eur J Nucl Med Mol Imaging. 2008;35(12):2227-34.

Kasai K, Yamasue H, Gilbertson MW, Shenton ME, Rauch SL, Pitman RK. Evidence for acquired pregenual anterior cingulate gray matter loss from a twin study of combat-related posttraumatic stress disorder. Biological Psychiatry. 2008;63(6):550-6.

Kasparek T, Prikryl R, Schwarz D, Kucerova H, Marecek R, Mikl M, et al. Gray matter morphology and the level of functioning in one-year follow-up of first-episode schizophrenia patients. Prog Neuropsychopharmacol Biol Psychiatry. 2009;33(8):1438-46.

Kassubek J, Juengling FD, Kioschies T, Henkel K, Karitzky J, Kramer B, et al. Topography of cerebral atrophy in early Huntington's disease: a voxel based morphometric MRI study. J Neurol Neurosurg Psychiatry. 2004;75(2):213-20.

Kawada R, Yoshizumi M, Hirao K, Fujiwara H, Miyata J, Shimizu M, et al. Brain volume and dysexecutive behavior in schizophrenia. Prog Neuropsychopharmacol Biol Psychiatry. 2009;33(7):1255-60.

Ke X, Hong S, Tang T, Zou B, Li H, Hang Y, et al. Voxel-based morphometry study on brain structure in children with high-functioning autism. Neuroreport. 2008;19(9):921-5.

Keller SS, Baker G, Downes JJ, Roberts N. Quantitative MRI of the prefrontal cortex and executive function in patients with temporal lobe epilepsy. Epilepsy Behav. 2009;15(2):186-95.

Keller SS, Cresswell P, Denby C, Wieshmann U, Eldridge P, Baker G, et al. Persistent seizures following left temporal lobe surgery are associated with posterior and bilateral structural and functional brain abnormalities. Epilepsy Res. 2007;74(2-3):131-9.

Keller SS, Wieshmann UC, Mackay CE, Denby CE, Webb J, Roberts N. Voxel based morphometry of grey matter abnormalities in patients with medically intractable temporal lobe epilepsy: effects of side of seizure onset and epilepsy duration. J Neurol Neurosurg Psychiatry. 2002;73(6):648-55.

Kim EJ, Rabinovici GD, Seeley WW, Halabi C, Shu H, Weiner MW, et al. Patterns of MRI atrophy in tau positive and ubiquitin positive frontotemporal lobar degeneration. J Neurol Neurosurg Psychiatry. 2007;78(12):1375-8.

Kim JH, Lee JK, Koh SB, Lee SA, Lee JM, Kim SI, et al. Regional grey matter abnormalities in juvenile myoclonic epilepsy: a voxel-based morphometry study. NeuroImage. 2007;37(4):1132-7.

Kim JJ, Lee MC, Kim J, Kim IY, Kim SI, Han MH, et al. Grey matter abnormalities in obsessive-compulsive disorder: statistical parametric mapping of segmented magnetic resonance images. The British journal of psychiatry : the journal of mental science. 2001;179:330-4.

Kim MJ, Hamilton JP, Gotlib IH. Reduced caudate gray matter volume in women with major depressive disorder. Psychiatry Research. 2008;164(2):114-22.

Kim S, Youn YC, Hsiung GY, Ha SY, Park KY, Shin HW, et al. Voxel-based morphometric study of brain volume changes in patients with Alzheimer's disease assessed according to the Clinical Dementia Rating score. J Clin Neurosci. 2011;18(7):916-21.

Kirchner H, Kremmyda O, Hufner K, Stephan T, Zingler V, Brandt T, et al. Clinical, electrophysiological, and MRI findings in patients with cerebellar ataxia and a bilaterally pathological head-impulse test. Ann N Y Acad Sci. 2011;1233:127-38.

Kobel M, Bechtel N, Specht K, Klarhofer M, Weber P, Scheffler K, et al. Structural and functional imaging approaches in attention deficit/hyperactivity disorder: does the temporal lobe play a key role? Psychiatry Research. 2010;183(3):230-6.

Koprivova J, Horacek J, Tintera J, Prasko J, Raszka M, Ibrahim I, et al. Medial frontal and dorsal cortical morphometric abnormalities are related to obsessive-compulsive disorder. Neuroscience letters. 2009;464(1):62-6.

Kostic VS, Agosta F, Petrovic I, Galantucci S, Spica V, Jecmenica-Lukic M, et al. Regional patterns of brain tissue loss associated with depression in Parkinson disease. Neurology. 2010;75(10):857-63.

Koutsouleris N, Gaser C, Jager M, Bottlender R, Frodl T, Holzinger S, et al. Structural correlates of psychopathological symptom dimensions in schizophrenia: a voxel-based morphometric study. NeuroImage. 2008;39(4):1600-12.

Kronbichler M, Wimmer H, Staffen W, Hutzler F, Mair A, Ladurner G. Developmental dyslexia: gray matter abnormalities in the occipitotemporal cortex. Human Brain Mapping. 2008;29(5):613-25.

Kubicki M, Shenton ME, Salisbury DF, Hirayasu Y, Kasai K, Kikinis R, et al. Voxel-based morphometric analysis of gray matter in first episode schizophrenia. NeuroImage. 2002;17(4):1711-9.

Kurth F, Narr KL, Woods RP, O'Neill J, Alger JR, Caplan R, et al. Diminished gray matter within the hypothalamus in autism disorder: a potential link to hormonal effects? Biological Psychiatry. 2011;70(3):278-82.

Kwon H, Ow AW, Pedatella KE, Lotspeich LJ, Reiss AL. Voxel-based morphometry elucidates structural neuroanatomy of high-functioning autism and Asperger syndrome. Dev Med Child Neurol. 2004;46(11):760-4.

Lai CH, Hsu YY, Wu YT. First episode drug-naive major depressive disorder with panic disorder: gray matter deficits in limbic and default network structures. Eur Neuropsychopharmacol. 2010;20(10):676-82.

Lai CH, Wu YT. Fronto-temporo-insula gray matter alterations of first-episode, drug-naive and very late-onset panic disorder patients. J Affect Disord. 2012;140(3):285-91.

Lasek K, Lencer R, Gaser C, Hagenah J, Walter U, Wolters A, et al. Morphological basis for the spectrum of clinical deficits in spinocerebellar ataxia 17 (SCA17). Brain : a journal of neurology. 2006;129(Pt 9):2341-52.

Lazaro L, Bargallo N, Castro-Fornieles J, Falcon C, Andres S, Calvo R, et al. Brain changes in children and adolescents with obsessive-compulsive disorder before and after treatment: a voxel-based morphometric MRI study. Psychiatry Research. 2009;172(2):140-6.

Lee HY, Tae WS, Yoon HK, Lee BT, Paik JW, Son KR, et al. Demonstration of decreased gray matter concentration in the midbrain encompassing the dorsal raphe nucleus and the limbic subcortical regions in major depressive disorder: an optimized voxel-based morphometry study. J Affect Disord. 2011;133(1-2):128-36.

Lehericy S, Hartmann A, Lannuzel A, Galanaud D, Delmaire C, Bienaimee MJ, et al. Magnetic resonance imaging lesion pattern in Guadeloupean parkinsonism is distinct from progressive supranuclear palsy. Brain : a journal of neurology. 2010;133(Pt 8):2410-25.

Leung KK, Lee TM, Wong MM, Li LS, Yip PS, Khong PL. Neural correlates of attention biases of people with major depressive disorder: a voxel-based morphometric study. Psychological Medicine. 2009;39(7):1097-106.

Li CT, Lin CP, Chou KH, Chen IY, Hsieh JC, Wu CL, et al. Structural and cognitive deficits in remitting and non-remitting recurrent depression: a voxel-based morphometric study. NeuroImage. 2010;50(1):347-56.

Li L, Chen S, Liu J, Zhang J, He Z, Lin X. Magnetic resonance imaging and magnetic resonance spectroscopy study of deficits in hippocampal structure in fire victims with recent-onset posttraumatic stress disorder. Can J Psychiatry. 2006;51(7):431-7.

Li M, Cui L, Deng W, Ma X, Huang C, Jiang L, et al. Voxel-based morphometric analysis on the volume of gray matter in bipolar I disorder. Psychiatry Research. 2011;191(2):92-7.

Libon DJ, McMillan C, Gunawardena D, Powers C, Massimo L, Khan A, et al. Neurocognitive contributions to verbal fluency deficits in frontotemporal lobar degeneration. Neurology. 2009;73(7):535-42.

Lin K, Jackowski AP, Carrete H, Jr., de Araujo Filho GM, Silva HH, Guaranha MS, et al. Voxel-based morphometry evaluation of patients with photosensitive juvenile myoclonic epilepsy. Epilepsy Res. 2009;86(2-3):138-45.

Lochhead RA, Parsey RV, Oquendo MA, Mann JJ. Regional brain gray matter volume differences in patients with bipolar disorder as assessed by optimized voxel-based morphometry. Biological Psychiatry. 2004;55(12):1154-62.

Lui S, Deng W, Huang X, Jiang L, Ouyang L, Borgwardt SJ, et al. Neuroanatomical differences between familial and sporadic schizophrenia and their parents: an optimized voxel-based morphometry study. Psychiatry Research. 2009;171(2):71-81.

Lukas C, Schols L, Bellenberg B, Rub U, Przuntek H, Schmid G, et al. Dissociation of grey and white matter reduction in spinocerebellar ataxia type 3 and 6: a voxel-based morphometry study. Neuroscience letters. 2006;408(3):230-5.

Lyoo IK, Kim MJ, Stoll AL, Demopulos CM, Parow AM, Dager SR, et al. Frontal lobe gray matter density decreases in bipolar I disorder. Biological Psychiatry. 2004;55(6):648-51.

Mainz V, Schulte-Ruther M, Fink GR, Herpertz-Dahlmann B, Konrad K. Structural brain abnormalities in adolescent anorexia nervosa before and after weight recovery and associated hormonal changes. Psychosom Med. 2012;74(6):574-82.

Marcelis M, Suckling J, Woodruff P, Hofman P, Bullmore E, van Os J. Searching for a structural endophenotype in psychosis using computational morphometry. Psychiatry Research. 2003;122(3):153-67.

Martino D, Di Giorgio A, D'Ambrosio E, Popolizio T, Macerollo A, Livrea P, et al. Cortical gray matter changes in primary blepharospasm: a voxel-based morphometry study. Mov Disord. 2011;26(10):1907-12.

Massana G, Serra-Grabulosa JM, Salgado-Pineda P, Gasto C, Junque C, Massana J, et al. Parahippocampal gray matter density in panic disorder: a voxel-based morphometric study. The American journal of psychiatry. 2003;160(3):566-8.

Massimo L, Powers C, Moore P, Vesely L, Avants B, Gee J, et al. Neuroanatomy of apathy and disinhibition in frontotemporal lobar degeneration. Dement Geriatr Cogn Disord. 2009;27(1):96-104.

Matsuda H, Kitayama N, Ohnishi T, Asada T, Nakano S, Sakamoto S, et al. Longitudinal evaluation of both morphologic and functional changes in the same individuals with Alzheimer's disease. J Nucl Med. 2002;43(3):304-11.

Matsumoto R, Ito H, Takahashi H, Ando T, Fujimura Y, Nakayama K, et al. Reduced gray matter volume of dorsal cingulate cortex in patients with obsessive-compulsive disorder: a voxel-based morphometric study. Psychiatry Clin Neurosci. 2010;64(5):541-7.

Mazere J, Prunier C, Barret O, Guyot M, Hommet C, Guilloteau D, et al. In vivo SPECT imaging of vesicular acetylcholine transporter using [(123)I]-IBVM in early Alzheimer's disease. NeuroImage. 2008;40(1):280-8.

McAlonan GM, Cheung V, Cheung C, Chua SE, Murphy DG, Suckling J, et al. Mapping brain structure in attention deficit-hyperactivity disorder: a voxel-based MRI study of regional grey and white matter volume. Psychiatry Research. 2007;154(2):171-80.

McAlonan GM, Cheung V, Cheung C, Suckling J, Lam GY, Tai KS, et al. Mapping the brain in autism. A voxel-based MRI study of volumetric differences and intercorrelations in autism. Brain : a journal of neurology. 2005;128(Pt 2):268-76.

McAlonan GM, Daly E, Kumari V, Critchley HD, van Amelsvoort T, Suckling J, et al. Brain anatomy and sensorimotor gating in Asperger's syndrome. Brain : a journal of neurology. 2002;125(Pt 7):1594-606.

McAlonan GM, Suckling J, Wong N, Cheung V, Lienenkaemper N, Cheung C, et al. Distinct patterns of grey matter abnormality in high-functioning autism and Asperger's syndrome. J Child Psychol Psychiatry. 2008;49(12):1287-95.

McIntosh AM, Job DE, Moorhead TW, Harrison LK, Forrester K, Lawrie SM, et al. Voxel-based morphometry of patients with schizophrenia or bipolar disorder and their unaffected relatives. Biological Psychiatry. 2004;56(8):544-52.

McMillan AB, Hermann BP, Johnson SC, Hansen RR, Seidenberg M, Meyerand ME. Voxel-based morphometry of unilateral temporal lobe epilepsy reveals abnormalities in cerebral white matter. NeuroImage. 2004;23(1):167-74.

Melzer TR, Watts R, MacAskill MR, Pitcher TL, Livingston L, Keenan RJ, et al. Grey matter atrophy in cognitively impaired Parkinson's disease. J Neurol Neurosurg Psychiatry. 2012;83(2):188-94.

Menghini D, Hagberg GE, Petrosini L, Bozzali M, Macaluso E, Caltagirone C, et al. Structural correlates of implicit learning deficits in subjects with developmental dyslexia. Ann N Y Acad Sci. 2008;1145:212-21.

Meppelink AM, de Jong BM, Teune LK, van Laar T. Regional cortical grey matter loss in Parkinson's disease without dementia is independent from visual hallucinations. Mov Disord. 2011;26(1):142-7.

Mesaros S, Rovaris M, Pagani E, Pulizzi A, Caputo D, Ghezzi A, et al. A magnetic resonance imaging voxel-based morphometry study of regional gray matter atrophy in patients with benign multiple sclerosis. Arch Neurol. 2008;65(9):1223-30.

Mezzapesa DM, Ceccarelli A, Dicuonzo F, Carella A, De Caro MF, Lopez M, et al. Whole-brain and regional brain atrophy in amyotrophic lateral sclerosis. AJNR Am J Neuroradiol. 2007;28(2):255-9.

Miettinen PS, Pihlajamaki M, Jauhiainen AM, Niskanen E, Hanninen T, Vanninen R, et al. Structure and function of medial temporal and posteromedial cortices in early Alzheimer's disease. Eur J Neurosci. 2011;34(2):320-30.

Migliaccio R, Agosta F, Rascovsky K, Karydas A, Bonasera S, Rabinovici GD, et al. Clinical syndromes associated with posterior atrophy: early age at onset AD spectrum. Neurology. 2009;73(19):1571-8.

Molina V, Galindo G, Cortes B, de Herrera AG, Ledo A, Sanz J, et al. Different gray matter patterns in chronic schizophrenia and chronic bipolar disorder patients identified using voxel-based morphometry. European Archives of Psychiatry and Clinical Neuroscience. 2011;261(5):313-22.

Molina V, Sanz J, Villa R, Perez J, Gonzalez D, Sarramea F, et al. Voxel-based morphometry comparison between first episodes of psychosis with and without evolution to schizophrenia. Psychiatry Research. 2010;181(3):204-10.

Moorhead TW, Job DE, Whalley HC, Sanderson TL, Johnstone EC, Lawrie SM. Voxel-based morphometry of comorbid schizophrenia and learning disability: analyses in normalized and native spaces using parametric and nonparametric statistical methods. NeuroImage. 2004;22(1):188-202.

Morgen K, Sammer G, Courtney SM, Wolters T, Melchior H, Blecker CR, et al. Evidence for a direct association between cortical atrophy and cognitive impairment in relapsing-remitting MS. NeuroImage. 2006;30(3):891-8.

Morgen K, Sammer G, Weber L, Aslan B, Muller C, Bachmann GF, et al. Structural brain abnormalities in patients with Parkinson disease: a comparative voxel-based analysis using T1-weighted MR imaging and magnetization transfer imaging. AJNR Am J Neuroradiol. 2011;32(11):2080-6.

Morrell MJ, Jackson ML, Twigg GL, Ghiassi R, McRobbie DW, Quest RA, et al. Changes in brain morphology in patients with obstructive sleep apnoea. Thorax. 2010;65(10):908-14.

Morrell MJ, McRobbie DW, Quest RA, Cummin AR, Ghiassi R, Corfield DR. Changes in brain morphology associated with obstructive sleep apnea. Sleep Med. 2003;4(5):451-4.

Mory SB, Betting LE, Fernandes PT, Lopes-Cendes I, Guerreiro MM, Guerreiro CA, et al. Structural abnormalities of the thalamus in juvenile myoclonic epilepsy. Epilepsy Behav. 2011;21(4):407-11.

Muhlau M, Gaser C, Ilg R, Conrad B, Leibl C, Cebulla MH, et al. Gray matter decrease of the anterior cingulate cortex in anorexia nervosa. The American journal of psychiatry. 2007;164(12):1850-7.

Muhlau M, Weindl A, Wohlschlager AM, Gaser C, Stadtler M, Valet M, et al. Voxel-based morphometry indicates relative preservation of the limbic prefrontal cortex in early Huntington disease. J Neural Transm. 2007;114(3):367-72.

Mummery CJ, Patterson K, Price CJ, Ashburner J, Frackowiak RS, Hodges JR. A voxel-based morphometry study of semantic dementia: relationship between temporal lobe atrophy and semantic memory. Annals of neurology. 2000;47(1):36-45.

Nagano-Saito A, Washimi Y, Arahata Y, Kachi T, Lerch JP, Evans AC, et al. Cerebral atrophy and its relation to cognitive impairment in Parkinson disease. Neurology. 2005;64(2):224-9.

Nardo D, Hogberg G, Looi JC, Larsson S, Hallstrom T, Pagani M. Gray matter density in limbic and paralimbic cortices is associated with trauma load and EMDR outcome in PTSD patients. Journal of Psychiatric Research. 2010;44(7):477-85.

Neckelmann G, Specht K, Lund A, Ersland L, Smievoll AI, Neckelmann D, et al. Mr morphometry analysis of grey matter volume reduction in schizophrenia: association with hallucinations. Int J Neurosci. 2006;116(1):9-23.

Nishio Y, Hirayama K, Takeda A, Hosokai Y, Ishioka T, Suzuki K, et al. Corticolimbic gray matter loss in Parkinson's disease without dementia. Eur J Neurol. 2010;17(8):1090-7.

Nugent AC, Milham MP, Bain EE, Mah L, Cannon DM, Marrett S, et al. Cortical abnormalities in bipolar disorder investigated with MRI and voxel-based morphometry. NeuroImage. 2006;30(2):485-97.

O'Muircheartaigh J, Vollmar C, Barker GJ, Kumari V, Symms MR, Thompson P, et al. Focal structural changes and cognitive dysfunction in juvenile myoclonic epilepsy. Neurology. 2011;76(1):34-40.

Obermann M, Yaldizli O, De Greiff A, Lachenmayer ML, Buhl AR, Tumczak F, et al. Morphometric changes of sensorimotor structures in focal dystonia. Mov Disord. 2007;22(8):1117-23.

Ortiz-Gil J, Pomarol-Clotet E, Salvador R, Canales-Rodriguez EJ, Sarro S, Gomar JJ, et al. Neural correlates of cognitive impairment in schizophrenia. The British journal of psychiatry : the journal of mental science. 2011;199(3):202-10.

Overmeyer S, Bullmore ET, Suckling J, Simmons A, Williams SC, Santosh PJ, et al. Distributed grey and white matter deficits in hyperkinetic disorder: MRI evidence for anatomical abnormality in an attentional network. Psychological Medicine. 2001;31(8):1425-35.

Padovani A, Borroni B, Brambati SM, Agosti C, Broli M, Alonso R, et al. Diffusion tensor imaging and voxel based morphometry study in early progressive supranuclear palsy. J Neurol Neurosurg Psychiatry. 2006;77(4):457-63.

Pail M, Brazdil M, Marecek R, Mikl M. An optimized voxel-based morphometric study of gray matter changes in patients with left-sided and right-sided mesial temporal lobe epilepsy and hippocampal sclerosis (MTLE/HS). Epilepsia. 2010;51(4):511-8.

Paillere-Martinot M, Caclin A, Artiges E, Poline JB, Joliot M, Mallet L, et al. Cerebral gray and white matter reductions and clinical correlates in patients with early onset schizophrenia. Schizophrenia Research. 2001;50(1-2):19-26.

Pantano P, Totaro P, Fabbrini G, Raz E, Contessa GM, Tona F, et al. A transverse and longitudinal MR imaging voxel-based morphometry study in patients with primary cervical dystonia. AJNR Am J Neuroradiol. 2011;32(1):81-4.

Pardini M, Huey ED, Cavanagh AL, Grafman J. Olfactory function in corticobasal syndrome and frontotemporal dementia. Arch Neurol. 2009;66(1):92-6.

Peinemann A, Schuller S, Pohl C, Jahn T, Weindl A, Kassubek J. Executive dysfunction in early stages of Huntington's disease is associated with striatal and insular atrophy: a neuropsychological and voxel-based morphometric study. J Neurol Sci. 2005;239(1):11-9.

Pell GS, Briellmann RS, Pardoe H, Abbott DF, Jackson GD. Composite voxel-based analysis of volume and T2 relaxometry in temporal lobe epilepsy. NeuroImage. 2008;39(3):1151-61.

Peng J, Liu J, Nie B, Li Y, Shan B, Wang G, et al. Cerebral and cerebellar gray matter reduction in first-episode patients with major depressive disorder: a voxel-based morphometry study. Eur J Radiol. 2011;80(2):395-9.

Pereira JB, Junque C, Marti MJ, Ramirez-Ruiz B, Bargallo N, Tolosa E. Neuroanatomical substrate of visuospatial and visuoperceptual impairment in Parkinson's disease. Mov Disord. 2009;24(8):1193-9.

Pereira JM, Williams GB, Acosta-Cabronero J, Pengas G, Spillantini MG, Xuereb JH, et al. Atrophy patterns in histologic vs clinical groupings of frontotemporal lobar degeneration. Neurology. 2009;72(19):1653-60.

Pomarol-Clotet E, Canales-Rodriguez EJ, Salvador R, Sarro S, Gomar JJ, Vila F, et al. Medial prefrontal cortex pathology in schizophrenia as revealed by convergent findings from multimodal imaging. Molecular Psychiatry. 2010;15(8):823-30.

Price G, Cercignani M, Chu EM, Barnes TR, Barker GJ, Joyce EM, et al. Brain pathology in first-episode psychosis: magnetization transfer imaging provides additional information to MRI measurements of volume loss. NeuroImage. 2010;49(1):185-92.

Prinster A, Quarantelli M, Lanzillo R, Orefice G, Vacca G, Carotenuto B, et al. A voxel-based morphometry study of disease severity correlates in relapsing-- remitting multiple sclerosis. Mult Scler. 2010;16(1):45-54.

Prinster A, Quarantelli M, Orefice G, Lanzillo R, Brunetti A, Mollica C, et al. Grey matter loss in relapsing-remitting multiple sclerosis: a voxel-based morphometry study. NeuroImage. 2006;29(3):859-67.

Pujol J, Soriano-Mas C, Alonso P, Cardoner N, Menchon JM, Deus J, et al. Mapping structural brain alterations in obsessive-compulsive disorder. Archives of general psychiatry. 2004;61(7):720-30.

Qiu L, Tian L, Pan C, Zhu R, Liu Q, Yan J, et al. Neuroanatomical circuitry associated with exploratory eye movement in schizophrenia: a voxel-based morphometric study. PLoS One. 2011;6(10):e25805.

Rabinovici GD, Seeley WW, Kim EJ, Gorno-Tempini ML, Rascovsky K, Pagliaro TA, et al. Distinct MRI atrophy patterns in autopsy-proven Alzheimer's disease and frontotemporal lobar degeneration. Am J Alzheimers Dis Other Demen. 2007;22(6):474-88.

Raji CA, Lopez OL, Kuller LH, Carmichael OT, Becker JT. Age, Alzheimer disease, and brain structure. Neurology. 2009;73(22):1899-905.

Rami L, Gomez-Anson B, Monte GC, Bosch B, Sanchez-Valle R, Molinuevo JL. Voxel based morphometry features and follow-up of amnestic patients at high risk for Alzheimer's disease conversion. Int J Geriatr Psychiatry. 2009;24(8):875-84.

Ramirez-Ruiz B, Marti MJ, Tolosa E, Gimenez M, Bargallo N, Valldeoriola F, et al. Cerebral atrophy in Parkinson's disease patients with visual hallucinations. Eur J Neurol. 2007;14(7):750-6.

Reetz K, Kleiman A, Klein C, Lencer R, Zuehlke C, Brockmann K, et al. CAG repeats determine brain atrophy in spinocerebellar ataxia 17: a VBM study. PLoS One. 2011;6(1):e15125.

Reetz K, Lencer R, Hagenah JM, Gaser C, Tadic V, Walter U, et al. Structural changes associated with progression of motor deficits in spinocerebellar ataxia 17. Cerebellum. 2010;9(2):210-7.

Remy F, Mirrashed F, Campbell B, Richter W. Verbal episodic memory impairment in Alzheimer's disease: a combined structural and functional MRI study. NeuroImage. 2005;25(1):253-66.

Riccitelli G, Rocca MA, Pagani E, Martinelli V, Radaelli M, Falini A, et al. Mapping regional grey and white matter atrophy in relapsing-remitting multiple sclerosis. Mult Scler. 2012;18(7):1027-37.

Riederer F, Lanzenberger R, Kaya M, Prayer D, Serles W, Baumgartner C. Network atrophy in temporal lobe epilepsy: a voxel-based morphometry study. Neurology. 2008;71(6):419-25.

Roebling R, Scheerer N, Uttner I, Gruber O, Kraft E, Lerche H. Evaluation of cognition, structural, and functional MRI in juvenile myoclonic epilepsy. Epilepsia. 2009;50(11):2456-65.

Rojas DC, Peterson E, Winterrowd E, Reite ML, Rogers SJ, Tregellas JR. Regional gray matter volumetric changes in autism associated with social and repetitive behavior symptoms. BMC Psychiatry. 2006;6:56.

Rosen HJ, Gorno-Tempini ML, Goldman WP, Perry RJ, Schuff N, Weiner M, et al. Patterns of brain atrophy in frontotemporal dementia and semantic dementia. Neurology. 2002;58(2):198-208.

Ruscheweyh R, Deppe M, Lohmann H, Stehling C, Floel A, Ringelstein EB, et al. Pain is associated with regional grey matter reduction in the general population. Pain. 2011;152(4):904-11.

Salgado-Pineda P, Baeza I, Perez-Gomez M, Vendrell P, Junque C, Bargallo N, et al. Sustained attention impairment correlates to gray matter decreases in first episode neuroleptic-naive schizophrenic patients. NeuroImage. 2003;19(2 Pt 1):365-75.

Salgado-Pineda P, Fakra E, Delaveau P, McKenna PJ, Pomarol-Clotet E, Blin O. Correlated structural and functional brain abnormalities in the default mode network in schizophrenia patients. Schizophrenia Research. 2011;125(2-3):101-9.

Salgado-Pineda P, Junque C, Vendrell P, Baeza I, Bargallo N, Falcon C, et al. Decreased cerebral activation during CPT performance: structural and functional deficits in schizophrenic patients. NeuroImage. 2004;21(3):840-7.

Salmond CH, Ashburner J, Connelly A, Friston KJ, Gadian DG, Vargha-Khadem F. The role of the medial temporal lobe in autistic spectrum disorders. Eur J Neurosci. 2005;22(3):764-72.

Salvadore G, Nugent AC, Lemaitre H, Luckenbaugh DA, Tinsley R, Cannon DM, et al. Prefrontal cortical abnormalities in currently depressed versus currently remitted patients with major depressive disorder. NeuroImage. 2011;54(4):2643-51.

Samuraki M, Matsunari I, Chen WP, Yajima K, Yanase D, Fujikawa A, et al. Partial volume effect-corrected FDG PET and grey matter volume loss in patients with mild Alzheimer's disease. Eur J Nucl Med Mol Imaging. 2007;34(10):1658-69.

Sanchez-Castaneda C, Rene R, Ramirez-Ruiz B, Campdelacreu J, Gascon J, Falcon C, et al. Correlations between gray matter reductions and cognitive deficits in dementia with Lewy Bodies and Parkinson's disease with dementia. Mov Disord. 2009;24(12):1740-6.

Santana MT, Jackowski AP, da Silva HH, Caboclo LO, Centeno RS, Bressan RA, et al. Auras and clinical features in temporal lobe epilepsy: a new approach on the basis of voxel-based morphometry. Epilepsy Res. 2010;89(2-3):327-38.

Sasayama D, Hayashida A, Yamasue H, Harada Y, Kaneko T, Kasai K, et al. Neuroanatomical correlates of attention-deficit-hyperactivity disorder accounting for comorbid oppositional defiant disorder and conduct disorder. Psychiatry Clin Neurosci. 2010;64(4):394-402.

Scheuerecker J, Meisenzahl EM, Koutsouleris N, Roesner M, Schopf V, Linn J, et al. Orbitofrontal volume reductions during emotion recognition in patients with major depression. Journal of psychiatry & neuroscience : JPN. 2010;35(5):311-20.

Schmidt-Wilcke T, Luerding R, Weigand T, Jurgens T, Schuierer G, Leinisch E, et al. Striatal grey matter increase in patients suffering from fibromyalgia--a voxel-based morphometry study. Pain. 2007;132 Suppl 1:S109-16.

Seeley WW, Crawford R, Rascovsky K, Kramer JH, Weiner M, Miller BL, et al. Frontal paralimbic network atrophy in very mild behavioral variant frontotemporal dementia. Arch Neurol. 2008;65(2):249-55.

Seidman LJ, Biederman J, Liang L, Valera EM, Monuteaux MC, Brown A, et al. Gray matter alterations in adults with attention-deficit/hyperactivity disorder identified by voxel based morphometry. Biological Psychiatry. 2011;69(9):857-66.

Seminowicz DA, Labus JS, Bueller JA, Tillisch K, Naliboff BD, Bushnell MC, et al. Regional gray matter density changes in brains of patients with irritable bowel syndrome. Gastroenterology. 2010;139(1):48-57 e2.

Senda J, Kato S, Kaga T, Ito M, Atsuta N, Nakamura T, et al. Progressive and widespread brain damage in ALS: MRI voxel-based morphometry and diffusion tensor imaging study. Amyotroph Lateral Scler. 2011;12(1):59-69.

Shah PJ, Ebmeier KP, Glabus MF, Goodwin GM. Cortical grey matter reductions associated with treatment-resistant chronic unipolar depression. Controlled magnetic resonance imaging study. The British journal of psychiatry : the journal of mental science. 1998;172:527-32.

Shapleske J, Rossell SL, Chitnis XA, Suckling J, Simmons A, Bullmore ET, et al. A computational morphometric MRI study of schizophrenia: effects of hallucinations. Cerebral Cortex. 2002;12(12):1331-41.

Shiino A, Watanabe T, Kitagawa T, Kotani E, Takahashi J, Morikawa S, et al. Different atrophic patterns in early- and late-onset Alzheimer's disease and evaluation of clinical utility of a method of regional z-score analysis using voxel-based morphometry. Dement Geriatr Cogn Disord. 2008;26(2):175-86.

Sigmundsson T, Suckling J, Maier M, Williams S, Bullmore E, Greenwood K, et al. Structural abnormalities in frontal, temporal, and limbic regions and interconnecting white matter tracts in schizophrenic patients with prominent negative symptoms. The American journal of psychiatry. 2001;158(2):234-43.

Silani G, Frith U, Demonet JF, Fazio F, Perani D, Price C, et al. Brain abnormalities underlying altered activation in dyslexia: a voxel based morphometry study. Brain : a journal of neurology. 2005;128(Pt 10):2453-61.

Sobanski T, Wagner G, Peikert G, Gruhn U, Schluttig K, Sauer H, et al. Temporal and right frontal lobe alterations in panic disorder: a quantitative volumetric and voxel-based morphometric MRI study. Psychological Medicine. 2010;40(11):1879-86.

Song SK, Lee JE, Park HJ, Sohn YH, Lee JD, Lee PH. The pattern of cortical atrophy in patients with Parkinson's disease according to cognitive status. Mov Disord. 2011;26(2):289-96.

Sonty SP, Mesulam MM, Thompson CK, Johnson NA, Weintraub S, Parrish TB, et al. Primary progressive aphasia: PPA and the language network. Annals of neurology. 2003;53(1):35-49.

Soriano-Mas C, Hernandez-Ribas R, Pujol J, Urretavizcaya M, Deus J, Harrison BJ, et al. Cross-sectional and longitudinal assessment of structural brain alterations in melancholic depression. Biological Psychiatry. 2011;69(4):318-25.

Spano B, Cercignani M, Basile B, Romano S, Mannu R, Centonze D, et al. Multiparametric MR investigation of the motor pyramidal system in patients with 'truly benign' multiple sclerosis. Mult Scler. 2010;16(2):178-88.

Stanfield AC, Moorhead TW, Job DE, McKirdy J, Sussmann JE, Hall J, et al. Structural abnormalities of ventrolateral and orbitofrontal cortex in patients with familial bipolar disorder. Bipolar disorders. 2009;11(2):135-44.

Steinbrink C, Vogt K, Kastrup A, Muller HP, Juengling FD, Kassubek J, et al. The contribution of white and gray matter differences to developmental dyslexia: insights from DTI and VBM at 3.0 T. Neuropsychologia. 2008;46(13):3170-8.

Suchan B, Busch M, Schulte D, Gronemeyer D, Herpertz S, Vocks S. Reduction of gray matter density in the extrastriate body area in women with anorexia nervosa. Behavioural brain research. 2010;206(1):63-7.

Summerfield C, Junque C, Tolosa E, Salgado-Pineda P, Gomez-Anson B, Marti MJ, et al. Structural brain changes in Parkinson disease with dementia: a voxel-based morphometry study. Arch Neurol. 2005;62(2):281-5.

Suzuki M, Nohara S, Hagino H, Kurokawa K, Yotsutsuji T, Kawasaki Y, et al. Regional changes in brain gray and white matter in patients with schizophrenia demonstrated with voxel-based analysis of MRI. Schizophrenia Research. 2002;55(1-2):41-54.

Suzuki Y, Kiyosawa M, Wakakura M, Mochizuki M, Ishii K. Gray matter density increase in the primary sensorimotor cortex in long-term essential blepharospasm. NeuroImage. 2011;56(1):1-7.

Szeszko PR, Christian C, Macmaster F, Lencz T, Mirza Y, Taormina SP, et al. Gray matter structural alterations in psychotropic drug-naive pediatric obsessive-compulsive disorder: an optimized voxel-based morphometry study. The American journal of psychiatry. 2008;165(10):1299-307.

Tae WS, Hong SB, Joo EY, Han SJ, Cho JW, Seo DW, et al. Structural brain abnormalities in juvenile myoclonic epilepsy patients: volumetry and voxel-based morphometry. Korean J Radiol. 2006;7(3):162-72.

Tae WS, Joo EY, Kim ST, Hong SB. Gray, white matter concentration changes and their correlation with heterotopic neurons in temporal lobe epilepsy. Korean J Radiol. 2010;11(1):25-36.

Takahashi R, Ishii K, Kakigi T, Yokoyama K, Mori E, Murakami T. Brain alterations and mini-mental state examination in patients with progressive supranuclear palsy: voxel-based investigations using f-fluorodeoxyglucose positron emission tomography and magnetic resonance imaging. Dement Geriatr Cogn Dis Extra. 2011;1(1):381-92.

Takahashi R, Ishii K, Miyamoto N, Yoshikawa T, Shimada K, Ohkawa S, et al. Measurement of gray and white matter atrophy in dementia with Lewy bodies using diffeomorphic anatomic registration through exponentiated lie algebra: A comparison with conventional voxel-based morphometry. AJNR Am J Neuroradiol. 2010;31(10):1873-8.

Tang Y, Wang F, Xie G, Liu J, Li L, Su L, et al. Reduced ventral anterior cingulate and amygdala volumes in medication-naive females with major depressive disorder: A voxel-based morphometric magnetic resonance imaging study. Psychiatry Research. 2007;156(1):83-6.

Tanskanen P, Ridler K, Murray GK, Haapea M, Veijola JM, Jaaskelainen E, et al. Morphometric brain abnormalities in schizophrenia in a population-based sample: relationship to duration of illness. Schizophrenia Bulletin. 2010;36(4):766-77.

Tavanti M, Battaglini M, Borgogni F, Bossini L, Calossi S, Marino D, et al. Evidence of diffuse damage in frontal and occipital cortex in the brain of patients with post-traumatic stress disorder. Neurol Sci. 2012;33(1):59-68.

Thivard L, Pradat PF, Lehericy S, Lacomblez L, Dormont D, Chiras J, et al. Diffusion tensor imaging and voxel based morphometry study in amyotrophic lateral sclerosis: relationships with motor disability. J Neurol Neurosurg Psychiatry. 2007;78(8):889-92.

Thomaes K, Dorrepaal E, Draijer N, de Ruiter MB, van Balkom AJ, Smit JH, et al. Reduced anterior cingulate and orbitofrontal volumes in child abuse-related complex PTSD. The Journal of clinical psychiatry. 2010;71(12):1636-44.

Tian L, Meng C, Yan H, Zhao Q, Liu Q, Yan J, et al. Convergent evidence from multimodal imaging reveals amygdala abnormalities in schizophrenic patients and their first-degree relatives. PLoS One. 2011;6(12):e28794.

Tir M, Delmaire C, le Thuc V, Duhamel A, Destee A, Pruvo JP, et al. Motor-related circuit dysfunction in MSA-P: Usefulness of combined whole-brain imaging analysis. Mov Disord. 2009;24(6):863-70.

Toal F, Daly EM, Page L, Deeley Q, Hallahan B, Bloemen O, et al. Clinical and anatomical heterogeneity in autistic spectrum disorder: a structural MRI study. Psychological Medicine. 2010;40(7):1171-81.

Togao O, Yoshiura T, Nakao T, Nabeyama M, Sanematsu H, Nakagawa A, et al. Regional gray and white matter volume abnormalities in obsessive-compulsive disorder: a voxel-based morphometry study. Psychiatry Research. 2010;184(1):29-37.

Torelli F, Moscufo N, Garreffa G, Placidi F, Romigi A, Zannino S, et al. Cognitive profile and brain morphological changes in obstructive sleep apnea. NeuroImage. 2011;54(2):787-93.

Treadway MT, Grant MM, Ding Z, Hollon SD, Gore JC, Shelton RC. Early adverse events, HPA activity and rostral anterior cingulate volume in MDD. PLoS One. 2009;4(3):e4887.

Tregellas JR, Shatti S, Tanabe JL, Martin LF, Gibson L, Wylie K, et al. Gray matter volume differences and the effects of smoking on gray matter in schizophrenia. Schizophrenia Research. 2007;97(1-3):242-9.

Tu CH, Niddam DM, Chao HT, Chen LF, Chen YS, Wu YT, et al. Brain morphological changes associated with cyclic menstrual pain. Pain. 2010;150(3):462-8.

Uchida RR, Del-Ben CM, Busatto GF, Duran FL, Guimaraes FS, Crippa JA, et al. Regional gray matter abnormalities in panic disorder: a voxel-based morphometry study. Psychiatry Research. 2008;163(1):21-9.

Valente AA, Jr., Miguel EC, Castro CC, Amaro E, Jr., Duran FL, Buchpiguel CA, et al. Regional gray matter abnormalities in obsessive-compulsive disorder: a voxel-based morphometry study. Biological Psychiatry. 2005;58(6):479-87.

Valet M, Gundel H, Sprenger T, Sorg C, Muhlau M, Zimmer C, et al. Patients with pain disorder show gray-matter loss in pain-processing structures: a voxel-based morphometric study. Psychosom Med. 2009;71(1):49-56.

van den Heuvel OA, Remijnse PL, Mataix-Cols D, Vrenken H, Groenewegen HJ, Uylings HB, et al. The major symptom dimensions of obsessive-compulsive disorder are mediated by partially distinct neural systems. Brain : a journal of neurology. 2009;132(Pt 4):853-68.

van Tol MJ, van der Wee NJ, van den Heuvel OA, Nielen MM, Demenescu LR, Aleman A, et al. Regional brain volume in depression and anxiety disorders. Archives of general psychiatry. 2010;67(10):1002-11.

Vartiainen N, Kallio-Laine K, Hlushchuk Y, Kirveskari E, Seppanen M, Autti H, et al. Changes in brain function and morphology in patients with recurring herpes simplex virus infections and chronic pain. Pain. 2009;144(1-2):200-8.

Vasic N, Walter H, Hose A, Wolf RC. Gray matter reduction associated with psychopathology and cognitive dysfunction in unipolar depression: a voxel-based morphometry study. J Affect Disord. 2008;109(1-2):107-16.

Venkatasubramanian G, Jayakumar PN, Gangadhar BN, Keshavan MS. Neuroanatomical correlates of neurological soft signs in antipsychotic-naive schizophrenia. Psychiatry Research. 2008;164(3):215-22.

Wagner A, Greer P, Bailer UF, Frank GK, Henry SE, Putnam K, et al. Normal brain tissue volumes after long-term recovery in anorexia and bulimia nervosa. Biological Psychiatry. 2006;59(3):291-3.

Wagner G, Koch K, Schachtzabel C, Reichenbach JR, Sauer H, Schlosser Md RG. Enhanced rostral anterior cingulate cortex activation during cognitive control is related to orbitofrontal volume reduction in unipolar depression. Journal of psychiatry & neuroscience : JPN. 2008;33(3):199-208.

Wang F, Kalmar JH, Womer FY, Edmiston EE, Chepenik LG, Chen R, et al. Olfactocentric paralimbic cortex morphology in adolescents with bipolar disorder. Brain : a journal of neurology. 2011;134(Pt 7):2005-12.

Wang J, Jiang T, Cao Q, Wang Y. Characterizing anatomic differences in boys with attention-deficit/hyperactivity disorder with the use of deformation-based morphometry. AJNR Am J Neuroradiol. 2007;28(3):543-7.

Waragai M, Okamura N, Furukawa K, Tashiro M, Furumoto S, Funaki Y, et al. Comparison study of amyloid PET and voxel-based morphometry analysis in mild cognitive impairment and Alzheimer's disease. J Neurol Sci. 2009;285(1-2):100-8.

Watson DR, Anderson JM, Bai F, Barrett SL, McGinnity TM, Mulholland CC, et al. A voxel based morphometry study investigating brain structural changes in first episode psychosis. Behavioural brain research. 2012;227(1):91-9.

Whitford TJ, Grieve SM, Farrow TF, Gomes L, Brennan J, Harris AW, et al. Progressive grey matter atrophy over the first 2-3 years of illness in first-episode schizophrenia: a tensor-based morphometry study. NeuroImage. 2006;32(2):511-9.

Whitwell JL, Jack CR, Jr., Boeve BF, Senjem ML, Baker M, Rademakers R, et al. Voxel-based morphometry patterns of atrophy in FTLD with mutations in MAPT or PGRN. Neurology. 2009;72(9):813-20.

Whitwell JL, Jack CR, Jr., Kantarci K, Weigand SD, Boeve BF, Knopman DS, et al. Imaging correlates of posterior cortical atrophy. Neurobiology of Aging. 2007;28(7):1051-61.

Whitwell JL, Josephs KA, Rossor MN, Stevens JM, Revesz T, Holton JL, et al. Magnetic resonance imaging signatures of tissue pathology in frontotemporal dementia. Arch Neurol. 2005;62(9):1402-8.

Whitwell JL, Sampson EL, Loy CT, Warren JE, Rossor MN, Fox NC, et al. VBM signatures of abnormal eating behaviours in frontotemporal lobar degeneration. NeuroImage. 2007;35(1):207-13.

Whitwell JL, Warren JD, Josephs KA, Godbolt AK, Revesz T, Fox NC, et al. Voxel-based morphometry in tau-positive and tau-negative frontotemporal lobar degenerations. Neurodegener Dis. 2004;1(4-5):225-30.

Wilke M, Kaufmann C, Grabner A, Putz B, Wetter TC, Auer DP. Gray matter-changes and correlates of disease severity in schizophrenia: a statistical parametric mapping study. NeuroImage. 2001;13(5):814-24.

Wilson LB, Tregellas JR, Hagerman RJ, Rogers SJ, Rojas DC. A voxel-based morphometry comparison of regional gray matter between fragile X syndrome and autism. Psychiatry Research. 2009;174(2):138-45.

Wilson SM, Brambati SM, Henry RG, Handwerker DA, Agosta F, Miller BL, et al. The neural basis of surface dyslexia in semantic dementia. Brain : a journal of neurology. 2009;132(Pt 1):71-86.

Wilson SM, Henry ML, Besbris M, Ogar JM, Dronkers NF, Jarrold W, et al. Connected speech production in three variants of primary progressive aphasia. Brain : a journal of neurology. 2010;133(Pt 7):2069-88.

Witthaus H, Kaufmann C, Bohner G, Ozgurdal S, Gudlowski Y, Gallinat J, et al. Gray matter abnormalities in subjects at ultra-high risk for schizophrenia and first-episode schizophrenic patients compared to healthy controls. Psychiatry Research. 2009;173(3):163-9.

Wolf RC, Sambataro F, Vasic N, Wolf ND, Thomann PA, Landwehrmeyer GB, et al. Longitudinal functional magnetic resonance imaging of cognition in preclinical Huntington's disease. Exp Neurol. 2011;231(2):214-22.

Wolf RC, Vasic N, Schonfeldt-Lecuona C, Ecker D, Landwehrmeyer GB. Cortical dysfunction in patients with Huntington's disease during working memory performance. Human Brain Mapping. 2009;30(1):327-39.

Wright IC, Ellison ZR, Sharma T, Friston KJ, Murray RM, McGuire PK. Mapping of grey matter changes in schizophrenia. Schizophrenia Research. 1999;35(1):1-14.

Xie S, Xiao JX, Gong GL, Zang YF, Wang YH, Wu HK, et al. Voxel-based detection of white matter abnormalities in mild Alzheimer disease. Neurology. 2006;66(12):1845-9.

Yamasue H, Kasai K, Iwanami A, Ohtani T, Yamada H, Abe O, et al. Voxel-based analysis of MRI reveals anterior cingulate gray-matter volume reduction in posttraumatic stress disorder due to terrorism. P Natl Acad Sci USA. 2003;100(15):9039-43.

Yang P, Wang PN, Chuang KH, Jong YJ, Chao TC, Wu MT. Absence of gender effect on children with attention-deficit/hyperactivity disorder as assessed by optimized voxel-based morphometry. Psychiatry Research. 2008;164(3):245-53.

Yaouhi K, Bertran F, Clochon P, Mezenge F, Denise P, Foret J, et al. A combined neuropsychological and brain imaging study of obstructive sleep apnea. J Sleep Res. 2009;18(1):36-48.

Yatham LN, Lyoo IK, Liddle P, Renshaw PF, Wan D, Lam RW, et al. A magnetic resonance imaging study of mood stabilizer- and neuroleptic-naive first-episode mania. Bipolar disorders. 2007;9(7):693-7.

Yoo HK, Kim MJ, Kim SJ, Sung YH, Sim ME, Lee YS, et al. Putaminal gray matter volume decrease in panic disorder: an optimized voxel-based morphometry study. Eur J Neurosci. 2005;22(8):2089-94.

Yoo SY, Roh MS, Choi JS, Kang DH, Ha TH, Lee JM, et al. Voxel-based morphometry study of gray matter abnormalities in obsessive-compulsive disorder. J Korean Med Sci. 2008;23(1):24-30.

Zahn R, Buechert M, Overmans J, Talazko J, Specht K, Ko CW, et al. Mapping of temporal and parietal cortex in progressive nonfluent aphasia and Alzheimer's disease using chemical shift imaging, voxel-based morphometry and positron emission tomography. Psychiatry Research. 2005;140(2):115-31.

Zamboni G, Huey ED, Krueger F, Nichelli PF, Grafman J. Apathy and disinhibition in frontotemporal dementia: Insights into their neural correlates. Neurology. 2008;71(10):736-42.

Zhang J, Tan Q, Yin H, Zhang X, Huan Y, Tang L, et al. Decreased gray matter volume in the left hippocampus and bilateral calcarine cortex in coal mine flood disaster survivors with recent onset PTSD. Psychiatry Research. 2011;192(2):84-90.

Zhang TJ, Wu QZ, Huang XQ, Sun XL, Zou K, Lui S, et al. Magnetization transfer imaging reveals the brain deficit in patients with treatment-refractory depression. J Affect Disord. 2009;117(3):157-61.

Zou K, Deng W, Li T, Zhang B, Jiang L, Huang C, et al. Changes of brain morphometry in first-episode, drug-naive, non-late-life adult patients with major depression: an optimized voxel-based morphometry study. Biological Psychiatry. 2010;67(2):186-8.
